# The molecular mechanism and therapeutic landscape of copper and cuproptosis in cancer

**DOI:** 10.1038/s41392-025-02192-0

**Published:** 2025-05-09

**Authors:** Ziyu Guo, Danyao Chen, Lei Yao, Yuming Sun, Daishi Li, Jiayuan Le, Yating Dian, Furong Zeng, Xiang Chen, Guangtong Deng

**Affiliations:** 1 Department of Dermatology, Xiangya Hospital, Central South University, Changsha, China; 2 National Engineering Research Center of Personalized Diagnostic and Therapeutic Technology, Changsha, China; 3 Furong Laboratory, Changsha, Hunan China; 4 Hunan Key Laboratory of Skin Cancer and Psoriasis, Hunan Engineering Research Center of Skin Health and Disease, Xiangya Hospital, Central South University, Changsha, China; 5 National Clinical Research Center for Geriatric Disorders, Xiangya Hospital, Changsha, China; 6 Department of Thoracic Surgery, Xiangya Hospital, Central South University, Changsha, Hunan China; 7 Department of Liver Surgery, Xiangya Hospital, Central South University, Changsha, Hunan China; 8 Department of Plastic and Cosmetic Surgery, Xiangya Hospital, Central South University, Changsha, Hunan China; 9 Department of Oncology, Xiangya Hospital, Central South University, Changsha, Hunan China

**Keywords:** Cancer therapy, Cancer

## Abstract

Copper, an essential micronutrient, plays significant roles in numerous biological functions. Recent studies have identified imbalances in copper homeostasis across various cancers, along with the emergence of cuproptosis, a novel copper-dependent form of cell death that is crucial for tumor suppression and therapeutic resistance. As a result, manipulating copper levels has garnered increasing interest as an innovative approach to cancer therapy. In this review, we first delineate copper homeostasis at both cellular and systemic levels, clarifying copper’s protumorigenic and antitumorigenic functions in cancer. We then outline the key milestones and molecular mechanisms of cuproptosis, including both mitochondria-dependent and independent pathways. Next, we explore the roles of cuproptosis in cancer biology, as well as the interactions mediated by cuproptosis between cancer cells and the immune system. We also summarize emerging therapeutic opportunities targeting copper and discuss the clinical associations of cuproptosis-related genes. Finally, we examine potential biomarkers for cuproptosis and put forward the existing challenges and future prospects for leveraging cuproptosis in cancer therapy. Overall, this review enhances our understanding of the molecular mechanisms and therapeutic landscape of copper and cuproptosis in cancer, highlighting the potential of copper- or cuproptosis-based therapies for cancer treatment.

## Introduction

Copper, an essential trace element crucial for various physiological processes, has garnered increasing attention for its intricate role in cancer biology.^1–4^ Intracellular copper levels are maintained within a strict range, with even slight elevations potentially causing cytotoxic effects and cell death, emphasizing the need for precise regulation of copper absorption, distribution, and excretion.^5–8^ In the broader physiological context, copper homeostasis in the human body is primarily maintained through several key processes, including intestinal absorption, vascular transport, hepatic storage, biliary excretion, and utilization and excretion by other organs.^9–11^ At the cellular level, copper homeostasis involves its uptake and subsequent distribution across various cellular compartments, such as the cytoplasm, mitochondria, Golgi apparatus, and nucleus.^12–14^ This intricate process encompasses the storage and flux of copper within these organelles, as well as its expulsion from the cell.^7,9,15^ The entire sequence of events is coordinated through copper acting as a catalytic cofactor in its redox chemistry, which entails complex interactions with various intracellular enzymes and proteins.^16^

In the context of cancer, dysregulation of copper metabolism exerts a dual effect on tumor progression. On one hand, cuproplasia, the term that refers to copper-driven cellular growth and proliferation, involves multiple cancer pathways and regulatory mechanisms to promote tumor progression,^17^ which could be further accelerated by copper-dependent metastasis, angiogenesis, and immune escape.^6,18–21^ On the other hand, copper inhibits tumor growth by participating in the regulation of cell death processes, including apoptosis, pyroptosis, necroptosis, ferroptosis, and autophagy, as well as by activating immune responses.^16,22–25^ In 2022, Tsvetkov et al. introduced the concept of cuproptosis, marking a new milestone in the study of copper-induced cell death mechanisms.^26^ Cuproptosis involves the binding of copper to lipoylated enzymes within the tricarboxylic acid cycle, which triggers protein aggregation, and proteotoxic stress, and ultimately leads to cell death.^26^

Given the burgeoning interest in copper metabolism and cuproptosis, it is crucial to develop a comprehensive understanding of the molecular mechanism and therapeutic landscape of copper and cuproptosis in cancer. In this review, we first delineate copper homeostasis and clarify both its protumorigenic and antitumorigenic functions in cancer. We then outline key milestones and molecular mechanisms of cuproptosis and explore its roles in cancer biology. Additionally, we summarize emerging therapeutic opportunities targeting copper and discuss the clinical associations of cuproptosis-related genes. Finally, we examine potential biomarkers for cuproptosis and discuss existing challenges and future prospects for leveraging cuproptosis in cancer therapy.

## Copper homeostasis in physiology

Copper overload or deficiency in the human body has been linked with a variety of diseases.^9,27^ Genetic mutations that disrupt copper homeostasis can lead to specific conditions such as Wilson’s disease (WD)^28,29^ and Menkes disease (MD).^30,31^ Copper imbalances are also associated with neurodegenerative diseases,^32^ cardiovascular diseases,^33,34^ and cancers,^2,3,35^ underscoring the critical importance of copper homeostasis in sustaining body health.^36^ Copper typically exists in biological systems in both copper (II) and copper (I) oxidation states.^37^ Copper (I) is predominantly found within the reducing environment of the cytoplasm, whereas copper (II) is more commonly in the oxidative conditions of the extracellular space.^4^ The highly oxidative and reductive nature imparts copper a dual role within physiological processes. On one hand, copper acts as a co-factor for numerous enzymes by facilitating electron transfer.^4,38^ On the other hand, excessive copper accumulation can disrupt cellular metabolism, potentially causing cellular damage or death.^39^ Thus, a strictly regulated copper homeostasis system is essential to ensure adequate copper levels for enzymatic functions while preventing toxic accumulation.^40,41^ Copper homeostasis encompasses the mechanisms of copper absorption, distribution, utilization, and excretion. This balance is maintained through a network of transporter proteins, chaperones, and storage molecules that orchestrate copper’s cellular entry, trafficking, enzyme incorporation, and elimination.^42^ We will outline the mechanisms of copper homeostasis in both systemic and cellular contexts.

## Systemic copper homeostasis

Copper is widely present throughout the human body, with a total amount of 100–200 mg.^43^ The concentrations of copper in organs and tissues vary, ranging from 3 mg (kidneys) to 46 mg (bone) in an adult weighing 70 kg.^44^ To maintain systemic copper homeostasis, a daily copper intake between 0.8 and 2.4 mg is recommended.^45^

**Fig. 1:**
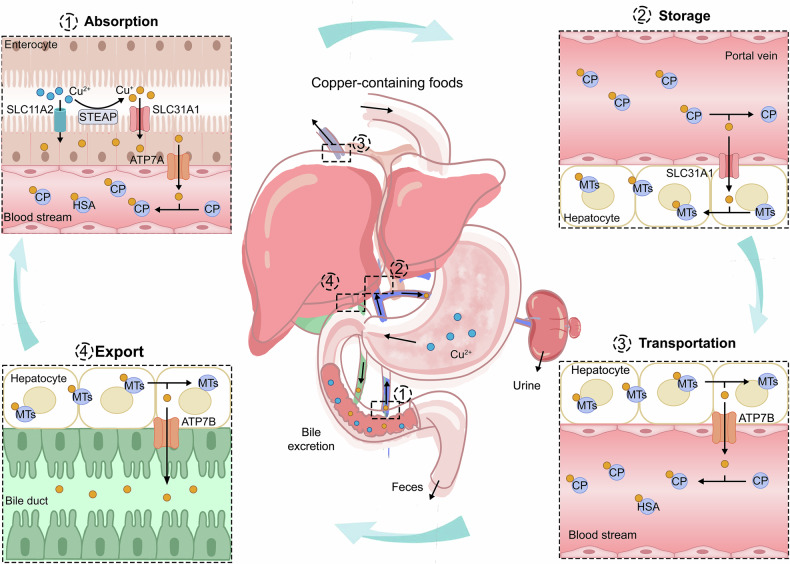
Systemic copper homeostasis. Systemic copper homeostasis involves intestinal absorption, hepatic storage, systemic transport, and biliary excretion. Dietary copper is predominantly absorbed in the small intestine, where copper (II) is reduced to copper (I) by members of the STEAP family. Copper (I) is then transported into enterocytes via SLC31A1, with a small amount entering through SLC11A2. Inside the intestinal epithelial cells, copper (I) binds to the copper chaperone and is transported to the basolateral side, where it is exported into the bloodstream via ATP7A. In the bloodstream, copper (I) binds to soluble copper chaperone proteins, primarily CP. Copper is transported to the liver through the portal vein, where hepatocytes uptake copper (I) from the bloodstream via SLC31A1. Within the hepatocytes, copper (I) can either be stored in MTs or re-enter the bloodstream through the ATP7B for distribution to other tissues. Excess copper is processed in the liver and excreted via bile, which is the primary route for copper elimination. Abbreviations: *STEAP* six-transmembrane epithelial antigen of the prostate, *SLC31A1* solute carrier family 1 member 1, *SLC11A2* solute carrier family 11 member 2, *ATP7A/B* ATPase copper transporter 7A and 7B, *CP* ceruloplasmin, *MT* metallothionein

Copper is mostly absorbed from dietary sources, including animal offal, seafood, and nuts,^46^ and it initially exists in the digestive system as copper (II)^10^ (Fig. 1). The primary site of dietary copper absorption is the small intestine, predominantly in the duodenum and jejunum regions,^47^ where copper (II) can permeate the cytoplasm via the nonspecific divalent metal transporter 1 (DMT1, also known as solute carrier family 11 member 2, SLC11A2).^48^ Additionally, members of the six-transmembrane epithelial antigen of the prostate (STEAP) family (including STEAP2, STEAP3, and STEAP4) and duodenal cytochrome b (DCYTB) function as copper reductases, ensuring that copper (II) is maintained in its reduced state copper (I) to facilitating cellular uptake.^9,49^ The entry of copper (I) into cells is primarily mediated by copper transporter 1 (CTR1, also known as SLC31A1) and CTR2 (SLC31A2), which are located at the apical membrane of intestinal epithelial cells, representing a major pathway for copper absorption.^50,51^ Notably, DMT1 has also been implicated in the uptake of copper (I), potentially serving as a compensatory mechanism in the absence of CTR1.^32,52^

The distribution of copper within the body can be divided into two phases. Dietary copper entering the bloodstream is initially transported to the liver and kidneys through the portal vein. Copper in the liver is then distributed to other tissues or organs via the blood circulatory system with the aid of circulating chaperone proteins.^53^ Specifically, after entering the intestinal epithelial cells, copper (I) binds to the copper chaperone antioxidant protein 1 (ATOX1) and is mainly transported to the opposite side of the epithelial cell, where it is exported into the bloodstream via copper-transporting P-type ATPases α (ATP7A).^54^ In the bloodstream, copper (II) predominantly binds to several copper chaperone proteins and is transported to the liver and kidneys. Approximately 75% of copper (II) binds to plasma ceruloplasmin (CP), 25% to human serum albumin, and about 0.2% to histidine and macroglobulin.^55,56^ The liver is the principal organ for copper storage and excretion, serving as the central regulatory mechanism for copper homeostasis. Within hepatocytes, copper is chelated by metallothionein isoforms 1 and 2 (MT1/2), facilitating its storage.^57^ MTs are thiol-rich reducing molecules that exhibit a high affinity for copper and are pivotal in maintaining copper equilibrium through the storage and timely release of excess copper.^58^ Excess copper is released into the bloodstream through the mediation of ATP7B, where it subsequently binds to the soluble chaperone proteins and is transported to specific tissues and organs.^59^ ATP7A and ATP7B, two subtypes of the P-type ATPase family, play a crucial role in copper export.^60^ ATP7A is predominantly expressed in the small intestine, whereas ATP7B is mainly found in the liver. Consequently, ATP7A is responsible for exporting copper from the intestines, while ATP7B facilitates copper export from the liver.

Copper, once released into the bloodstream from the liver, is transported to specific tissues and organs such as the brain, heart, muscles, and bones for utilization.^37,61^ In these organs, copper catalyzes a range of essential physiological processes, such as maintenance of redox homeostasis, mitochondrial energy production, remodeling of the extracellular matrix (ECM), and metabolism of tyrosine and neurotransmitters.^9,62–64^ For instance, copper participates in myelination and interacts with synaptic proteins and neurotransmitter receptors, highlighting its significant function at synapses.^32,65^ Excretion of excess copper occurs primarily through the following pathways: *(I)* metabolized copper in the liver is incorporated into bile and subsequently excreted from the body in the form of secretory vesicles, which represents the primary mechanism for endogenous copper elimination;^66^ *(II)* unabsorbed copper in the intestinal tract is excreted through feces;^67^ *(III)* a small amount of copper (10–50 µg/day) is also eliminated through urine via the kidneys;^8^ and *(IV)* sweat and menstruation also contribute to copper excretion.^5^ In instances of high copper intake, the human body mainly regulates copper homeostasis by increasing bile excretion or reducing absorption, and vice versa.^67^

## Intracellular copper homeostasis

**Fig. 2:**
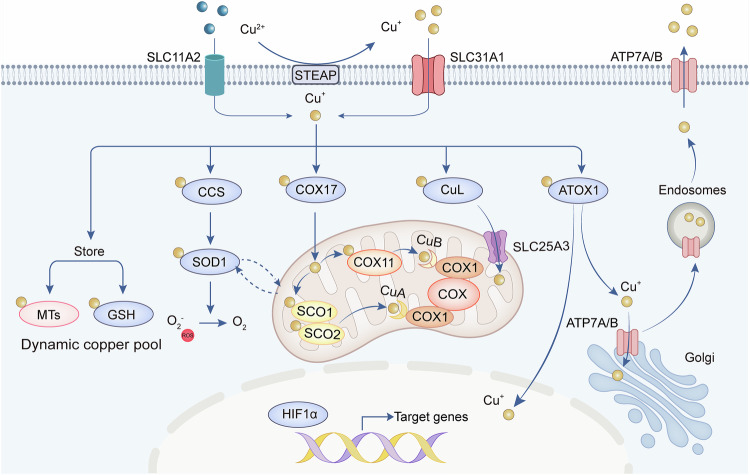
Intracellular copper homeostasis. Within the cell, copper (I) can be sequestered by MTs and GSH, forming a dynamic copper pool, or it can bind to copper chaperone proteins, including CCS, COX17, CuL, and ATOX1, for transport to various organelles. CCS delivers copper (I) to the SOD1, facilitating the conversion of superoxide radicals into oxygen and thereby protecting the cell from oxidative stress. COX17 transports copper (I) to mitochondrial SCO1 and COX11, essential for COX assembly. CuL binds to copper in the cytosol and triggers copper transport into the mitochondria via SLC25A3. ATOX1 directs copper (I) to ATP7A/B in the TGN. When there is an excess of copper, ATP7A/B translocates to vesicular compartments and fuses with the plasma membrane to expel the excess copper. Also, CCS and ATOX1 are involved in transporting copper to the cell nucleus, which is crucial for activating various transcription factors. Abbreviations: *MT* metallothionein, *GSH* glutathione, *CCS* copper chaperone for superoxide dismutase, *COX* cytochrome oxidase, *SCO1* synthesis of cytochrome c oxidase 1, *CuL* copper ligands, *ATOX1* antioxidant protein 1, *SOD1* superoxide dismutase 1, *ATP7A/B* ATPase copper transporter 7A and 7B, *TGN* trans-Golgi network

Once entering the cell, copper is intricately coordinated by a fine-tuning network, involving the intricmate crosstalk between cytoplasm and different organelles (Fig. 2). The intracellular copper form includes two forms: a tightly bound protein pool at the micromolar level, and a bioavailable, labile pool at the femtomolar level.^68^ Although the concentration of free copper in cells is almost negligible, it still has a potentially detrimental effect on cell membranes, proteins, and nucleic acids. Therefore, cellular copper homeostasis is rigorously regulated to maintain copper levels within a specific and narrow range.^41^

Within the cytoplasm, superoxide dismutase (CCS), a soluble copper chaperone protein, immediately delivers copper (I) to the copper-binding site of superoxide dismutase 1 (SOD1).^69,70^ SOD1 is a principal antioxidant enzyme located in the cytosol and the mitochondrial intermembrane space (IMS), where it catalyzes the conversion of superoxide radicals to hydrogen peroxide and oxygen, thereby protecting cells from oxidative stress damage.^71^ The distribution of SOD1 between the cytosol and IMS is regulated by CCS, which mediates the formation of disulfide bonds in SOD1.^72,73^ This is crucial for its proper spatial conformation and enzymatic activity.^74^ Intracellular copper typically binds to MTs and non-proteinaceous ligands such as glutathione (GSH), which provide storage and detoxifying functions.^75^ The regulation of copper ion concentration is also mediated by the expression of MTs, which is increased in response to elevated copper levels.^76,77^ This mechanism ensures that the cell can adapt to fluctuating copper availability while maintaining essential biological processes.

Mitochondria are the major organelle for the storage and utilization of copper, playing a crucial role in cellular copper homeostasis.^49,78^ The copper chaperone COX17 facilitates the transport of copper to the mitochondria by shuttling between the cytoplasm and the mitochondria. Copper ligands (CuL), a non-proteinaceous and low molecular weight complex, are also involved in copper transport.^17^ Specifically, CuL binds to copper in the cytosol and triggers copper transport into the mitochondria through transporters such as SLC25A3.^79,80^ Copper is a fundamental element in mitochondria function, particularly through its role with cytochrome oxidase (COX), the enzyme complex that drives oxidative phosphorylation (OXPHOS).^81^ The COX complex, also known as mitochondrial respiratory chain complex IV and cytochrome C oxidase (CCO), requires copper and heme as essential cofactors to facilitate the process of ATP production by transferring electrons through the respiratory chain to molecular oxygen. Specifically, copper is involved in the assembly of the COX complex, which consists of the two core subunits COX1 and COX2, through two distinct pathways.^70,82^ On one hand, COX17 binds to and delivers Cu to the synthesis of cytochrome c oxidase 1 (SCO1) or SCO2, which then transfers the Cu to the CuA site in the core subunit of COX2. SCO1 particularly helps in the copper metallation of COX2 by binding copper on the intermembrane space side and inserting it into COX2. On the other hand, COX17 binds to and transfers copper to COX11, which conveys copper to the CuB site of COX1. As COX17 serves as a primary copper donor in the IMS,^83^ mutations in COX17 can reduce CCO activity and cause mitochondrial dysfunction and oxidative stress,^84^ further supporting copper’s significance in mitochondria.

The Golgi apparatus works as the central compartment for copper homeostasis.^85–87^ ATP7A and ATP7B are the primary transport proteins for exporting cellular copper, and their localization and function are vital for regulating copper homeostasis.^88^ Under physiological copper levels, ATP7A/B are situated in the TGN, where they pump copper into the lumen of the TGN by the copper chaperone ATOX1.^89^ When there is an excess of copper within the cell, ATP7A and ATP7B can relocate to vesicular compartments and fuse with the plasma membrane to expel excess copper, thus preventing copper toxicity.^90^ This relocation is essential for modulating copper efflux. Once copper levels return to physiological norms, ATP7A and ATP7B are recycled back to the TGN,^60^ where copper facilitates the synthesis of copper enzymes including tyrosinase, lysyl oxidase (LOX), CP, and SOD3.^91^ These enzymes are integral to various biological processes such as connective tissue development, iron metabolism, and melanin production.^92–94^

Copper also plays a pivotal role in the nucleus.^32^ Specifically, CCS and ATOX1 are both involved in transporting copper to the cell nucleus, where it is essential for activating different transcription factors.^95–97^ In human hepatocellular carcinoma (HCC) cells, the expression of genes induced under low oxygen conditions relies on the presence of CCS and copper. These components are critical for enabling hypoxia-inducible factor 1α (HIF1α) to bind to both the transcriptional co-activator protein p300 and to elements in target genes that respond to hypoxia.^98^ Moreover, ATOX1 carries copper into the nucleus and functions as a novel transcription factor, thereby contributing to cell proliferation.^96^

## Copper functions in cancer

Extensive research has revealed unique metabolic patterns of copper in different cancers.^17,27,99^ Compared to normal tissues, tumor tissues exhibit a higher demand for copper.^2,100^ For instance, elevated levels of copper in the tumor tissue and serum have been observed in patients with oral,^101^ thyroid,^102^ breast,^103,104^ lung,^105,106^ pancreatic,^107^ gallbladder,^108^ colorectal,^109^ prostate,^110^ and gynecological cancers,^111^ and are significantly associated with poor prognostic outcomes. This increased copper demand is primarily because copper is required as a cofactor for multiple enzymes involved in cellular energy metabolism (such as CCO) and antioxidant defenses (such as SOD), thereby meeting the substantial energy needs of rapidly dividing tumor cells.^112^ Additionally, copper can also negatively affect tumors due to its redox activity and improper binding with functional macromolecules.^26,113^ Therefore, an imbalance in copper homeostasis could play a dual role, in promoting or suppressing tumors in various contexts.

## Tumor-promoting functions of copper

### Copper and cuproplasia

**Fig. 3:**
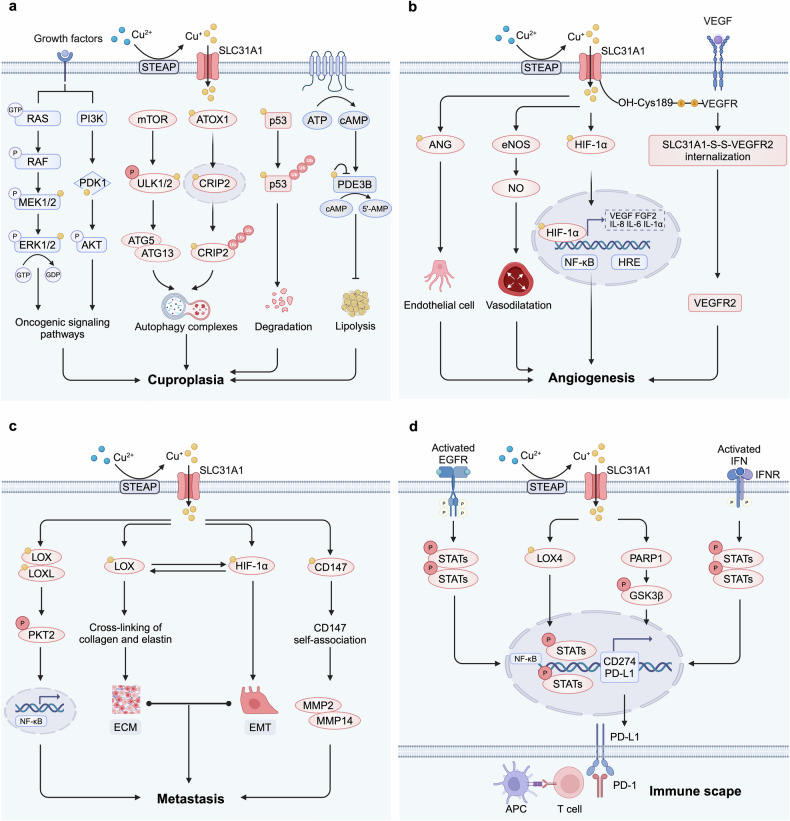
Mechanisms of copper in promoting tumor. **a** Copper binds to MEK1/2 and PDK1, activating oncogenic signaling pathways. It also activates ULK1/2 or enters the nucleus to induce the degradation of CRIP2, promoting autophagy. Additionally, copper interacts with the p53 protein, leading to its degradation. Copper also inhibits PDE3B, promoting lipolysis, which collectively contributes to cuproplasia. **b** Copper directly binds or activates angiogenic factors such as ANG and NO and interacts with HIF-1 to enhance NF-κB activity, promoting the expression of angiogenic mediators. Disulfide bonds formed between CTR1 and VEGFR2 activate VEGFR2 signaling, facilitating angiogenesis. **c** Copper promotes the expression of LOX/LOXL and HIF-1α, synergistically enhancing tumor metastasis through a positive feedback mechanism. Copper also binds to CD147 to promote its self-association, further enhancing metastasis. **d** Copper upregulates the expression of PD-L1 in cancer cells through multiple pathways, inhibiting T lymphocytes and inducing exhaustion, thereby facilitating immune escape. Created by BioRender. Abbreviations: ULK1/2 unc-51-like autophagy activating kinases 1 and 2, CRIP2 copper-binding protein cysteine-rich protein 2, PDE3B phosphodiesterase 3B, ANG angiogenin, NO nitric oxide, HIF-1 hypoxia-Inducible Factor-1, CTR1 copper transporter 1, VEGFR vascular endothelial growth factor receptor, LOX lysyl oxidase, LOXL lysyl oxidase-like protein, HIF-1α hypoxia-inducible factor-1α, PD-L1 programmed death-ligand 1

Copper, as a transition metal element and essential nutrient, plays diverse and critical roles in oncology.^114^ To better elucidate the association between copper and cancer, researchers coined the term “cuproplasia” in 2022, which is described as copper-dependent cellular growth and proliferation and exemplifies “metalloplasias”.^17^ The process encompasses the interactions of copper with various cellular mechanisms, including kinase signaling pathways, autophagy, the ubiquitin-proteasome system (UPS), epigenetic regulation, and metabolic pathways (Fig. 3a).

Copper activates a series of cancer-related kinase-signaling pathways, including the RAS-RAF-MEK-ERK1/2 and PI3K-PDK1-AKT pathways.^115,116^ Specifically, copper acts as a cofactor of MEK1/2 can allosterically enhance its capability to phosphorylate ERK1/2 in a dose-dependent manner, which further promotes the expression of c-myc, c-fos, and c-jun in the nucleus to regulate tumor growth.^117–119^ This copper-dependent vulnerability has been demonstrated in tumor models with BRAF or KRAS mutations. Mutations in BRAF, most commonly BRAF^V600E^, drive the development of various cancers, including melanoma, lung cancer, and thyroid cancer, and its carcinogenic signal transmission requires copper to bind to MEK and facilitate the activation of ERK1/2.^120,121^ Disruption of copper availability, either through the genetic ablation of the CTR1 or by introducing mutations that impair copper binding in the surface-accessible regions of MEK1, can decrease the signal transduction and tumorigenesis driven by BRAF.^121^ In the KRAS^G12V^-mutated colorectal cancer (CRC) model, tumor cells can obtain copper to promote cell growth through an atypical mechanism involving micropinocytosis. Upregulation of ATP7A or exposure to the copper chelator tetrathiomolybdate (TTM) results in reduced phosphorylation of ERK1/2, indicating that copper bioavailability represents a KRAS-selective vulnerability.^122^ Additionally, copper can also bind to the H117 and H203 sites on PDK1, enhancing its interaction with AKT and activating AKT-mediated oncogenic signaling. By depleting CTR1 or using copper chelators to inhibit the copper axis, AKT signaling can be reduced, thereby decreasing tumorigenesis and cell proliferation.^123^

Copper stimulates autophagy to regulate cuproplasia in response to various stressors such as nutritional starvation, metabolic imbalance, hypoxia, oxidative stress, and oncogene activation. Cancer cells generate nutrients and energy through a cellular process known as autophagy, supporting the survival and proliferation of the tumor cells.^124–126^ Unc-51-like autophagy activating kinases 1 and 2 (ULK1/2), downstream targets of the major nutrient-sensing kinase mechanistic target of rapamycin complex 1 (mTORC1), serve as pivotal regulators within the autophagy pathway.^126^ The direct binding of copper to ULK1/2 is necessary for kinase activity and serves as a regulatory factor that promotes the phosphorylation and activation of the autophagy proteins ATG7 and ATG13, leading to the formation of the autophagy complexes and ultimately enhancing tumor growth.^127^ Fluctuations in intracellular copper concentrations can accordingly regulate ULK1/2 kinase activity.^128^ The absence of the CTR1 gene or mutations in ULK1/2 can impair its copper-binding capacity, thereby inhibiting copper-ULK1/2 dependent downstream signaling and the formation of autophagy complexes, reducing cancer cell proliferation and increasing sensitivity to “nutrient starvation”.^128^ Furthermore, copper can activate autophagy by binding to the nuclear copper-binding protein cysteine-rich protein 2 (CRIP2).^129^ Mechanistically, copper is transferred to the nucleus via ATOX1, binds to CRIP2, inducing changes in the secondary structure of the CRIP2 protein, and promotes its ubiquitin-mediated proteasome degradation. This process leads to ROS-mediated activation of autophagy.^129^

Copper activates the UPS to regulate the degradation of certain proteins, such as p53 and XIAP, thereby promoting the proliferation and growth of tumor cells. The tumor suppressor protein p53, encoded by the critical tumor suppressor gene TP53, plays a pivotal role in regulating cell growth. Mutations in TP53 can lead to functional impairments that promote uncontrolled cell proliferation and the development of cancer.^130,131^ Intracellular free zinc binds to p53 to ensure its activity and stability,^132^ which could be displaced by copper, leading to aberrant folding of the protein and subsequent degradation via the UPS.^133^ Specifically, copper promotes p53 degradation through positive allosteric activation of the E2 conjugating enzyme branch UBE2D1-UBE2D4.^134^ The X-linked inhibitor of apoptosis protein (XIAP) is a potent anti-apoptotic factor belonging to the inhibitor of apoptosis (IAP) family, capable of directly inhibiting caspases and regulating cell apoptosis.^135^ XIAP exhibits strong copper affinity, and reversible binding with copper leads to its rapid ubiquitination and subsequent proteasomal degradation, thereby reducing its anti-apoptotic inhibitory capacity.^136^

Copper inhibits several key mitochondrial enzymes, disrupting the production of metabolites involved in epigenetic regulation.^137–139^ For example, copper inhibits pyruvate dehydrogenase, which in turn suppresses the conversion of pyruvate to acetyl-CoA, a metabolite required for histone acetylation by histone acetyltransferases (HATs) and global transcription.^140^ The acetylation levels of histones are jointly regulated by HATs and histone deacetylases.^141^ Decreased acetylation can lead to increased proliferation and differentiation of tumor cells. In human hepatoma Hep3B cells, exposure to copper (II) leads to a reduction in histone H3 and H4 acetylation by inhibiting the activity of HATs.^142^ Additionally, inhibition of COX17 leads to elevated mitochondrial copper levels in leukemic stem cells, which results in reduced cellular levels of S-adenosylmethionine, subsequently decreasing global DNA methylation and increasing chromatin accessibility, which contributes to promoting the differentiation of acute myeloid leukemia cells while reducing stem cell-like characteristics.^143^

Copper modulates tumor metabolism by interacting with molecules involved in lipid metabolic pathways. Cancer cells facilitate their proliferation and metastasis by harnessing energy from lipid catabolism.^144^ The classic 3’,5’-cyclic AMP (cAMP) pathway, which is essential for the breakdown of triglycerides into fatty acids and glycerol, plays a crucial role in lipid metabolism within the body.^145^ Copper serves as an endogenous regulator by binding to a key conserved cysteine residue within the active site of phosphodiesterase 3B and inhibiting its activity. This inhibition prevents the degradation of cAMP, thereby promoting cAMP-dependent lipolysis.^146^

### Copper and angiogenesis

Angiogenesis is a crucial process for the transport of oxygen and nutrients to tumors, facilitating tumor progression.^147,148^ Copper acts as a pivotal “switch” in angiogenic signaling by activating a multitude of pro-angiogenic and inflammatory factors^100,149–151^ (Fig. 3b). For example, copper binds directly to angiogenin (ANG), modulating its affinity towards endothelial cells and vascular smooth muscle cells, thereby promoting angiogenesis.^152,153^ Moreover, intracellular copper can stabilize the biochemical structure of transcription factor HIF-1 and enhance the activity of the nuclear factor NF-κB, thereby ensuring their transcriptional activity on angiogenesis genes such as VEGF and ceruloplasmin and promoting the expression of angiogenic mediators including FGF2, VEGF, IL-8, IL-6, and IL-1α.^154–156^ Copper also elevates endothelial nitric oxide synthase levels, thereby increasing the production of nitric oxide (NO). The increase in NO not only promotes vasodilation but also activates pro-tumor angiogenic signaling pathways.^157^

Copper homeostasis-associated proteins are also involved in copper-mediated angiogenesis. For example, CTR1 promotes angiogenesis by regulating the entry of copper into endothelial cells, activating a multitude of pro-angiogenic and inflammatory factors.^158^ CTR1 also transmits ROS signals induced by VEGF through the sulfenylation of cysteine 189 (Cys189) located at its C-terminal and promotes the formation of disulfide bonds between CTR1 and VEGFR2. The CTR1-VEGFR2 complex drives their co-endocytosis, activating sustained VEGFR2 signaling within endosomes, which is crucial for angiogenesis.^19^ Moreover, the copper-dependent transcription factor ATOX1 contributes to tumor angiogenesis and vascular remodeling by modulating the platelet-derived growth factor signaling pathway and activating the ATP7A-mediated LOX signaling pathway.^159,160^ Concurrently, ATP7A plays a critical role by inhibiting the autophagy-mediated degradation of VEGFR2, thus enhancing VEGFR2 signaling and further promoting angiogenesis.^160^ Additionally, the enzyme Amine oxidase copper-containing 3 (AOC3) has been shown to induce angiogenesis through a mechanism involving IL-1β-driven M2 macrophage infiltration.^161^ These interconnected mechanisms highlight the potential of targeting copper homeostasis as an anti-angiogenic therapeutic strategy for cancer.

### Copper and metastasis

Tumor metastasis is a dynamic and complex process in which copper plays a significant role^162,163^ (Fig. 3c). On one hand, copper-mediated cell proliferation and angiogenesis serve as fundamental components of this process.^164^ On the other hand, copper activates enzymes and signaling cascades related to metastasis, regulating the remodeling of the ECM and epithelial-mesenchymal transition (EMT), both of which are key pathways facilitating the metastasis of cancer.^164–166^

LOX and LOX-like (LOXL) isoforms are copper-dependent metalloenzymes that have been established as contributors to cancer cell metastasis.^167,168^ Intracellular copper accumulation can activate LOX/LOXL, promoting the cross-linking of collagen and elastin in the ECM, thereby creating a microenvironment conducive to tumor cell metastasis.^169–171^ The activation of LOX/LOXL stimulates signaling pathways involving protein kinase C α and protein tyrosine kinase 2, further driving the activation of transcription factors associated with cancer cell proliferation and metastasis.^172,173^ Furthermore, LOX promotes the cross-linking of ECM components such as elastin and collagen through the copper-dependent oxidoreductase cell motility mediator 1, which influences cellular activities and leads to alterations in the cytoskeletal proteins, enhancing extracellular migration and metastasis.^174,175^ Notably, Copper ions can induce the secretion of LOX by activating HIF-1α,^176^ while LOX enhances the synthesis of HIF-1α protein through a positive feedback mechanism, with both factors working synergistically to regulate and promote tumor progression.^177,178^ Copper also activates the interaction between HIF-1α and hypoxia-responsive elements as well as the HIF1α-Snail/Twist signaling pathway, promoting the development of EMT.^179,180^ In triple-negative breast cancer (TNBC), cells expressing SOX2^+^/OCT4^+^ exhibit significant metastatic potential due to copper-mediated activation of the AMPK/mTORC1 signaling pathway.^181^

The interaction between certain proteins and copper also plays a role in tumor metastasis. CD147 acts as a receptor for extracellular copper ions.^182^ In HCC cells, copper (II) binds to the proximal extramembrane domain of CD147 and mediates its self-association. This process activates the PI3K/AKT signaling pathway, leading to the upregulation of MMP-2 and MMP-14, which enhances the invasiveness of HCC cells.^182^ Copper chaperone proteins also play a key role in linking copper homeostasis with cancer metastasis. ATOX1 mediates the metastasis of breast cancer cells through coordinated copper transport along the ATP7A-LOX axis,^183^ making the levels of ATOX1 in tumor cells a potential predictive marker of metastatic potential and a biomarker for copper depletion therapy. Furthermore, the Secreted protein acidic and rich in cysteine, also known as osteonectin, contains a copper-binding domain.^184^ The binding of this domain to copper ions has been demonstrated to regulate cell-matrix interactions and enhance the metastasis of tumor cells.^185^

### Copper and immune escape

The interaction between programmed cell death protein 1 (PD-1) and its ligand PD-L1 is a core mechanism for tumor immune escape.^186–188^ Copper can upregulate the expression of PD-L1 in cancer cells through multiple pathways, inhibiting T lymphocytes and inducing exhaustion to facilitate immune escape (Fig. 3d). (1) Copper increases PD-L1 levels by inhibiting UPS-mediated degradation, facilitating the phosphorylation of STAT3 and EGFR, which are crucial proteins for tumor growth and immune escape.^20,189^ Copper chelators have also been shown to enhance the effectiveness of immune cells such as CD8^+^ T cells and natural killer (NK) cells in the tumor microenvironment (TME), thereby slowing tumor growth and improving survival rates in mice.^20^ (2) Copper promotes the secretion of LOXL4 by tumor cells. Exposure to LOXL4 results in macrophages within the TME adopting an immunosuppressive phenotype primarily mediated by interferon (IFN)-dependent signal transduction, which leads to transcription-dependent PD-L1 activation. The increased PD-L1 expression further impairs CD8^+^ T-cell function, promoting tumor immune escape and supporting tumor growth.^190^ (3) Copper ions contribute to tumor immune escape by upregulating the expression of CD274/PD-L1 (CD274 molecule), which serves as an immune checkpoint in cancer cells.^191^ (4) The disulfiram (DSF) and copper (DSF/Cu) complex upregulates PD-L1 expression by inhibiting poly (ADP-ribose) polymerase 1 (PARP1) activity and enhancing phosphorylation of glycogen synthase kinase-3β (GSK-3β) at Ser9 site, ultimately suppressing T cell infiltration.^192^

## Tumor-suppressing functions of copper

### Copper and apoptosis

Apoptosis is a strictly regulated form of PCD, characterized by distinct morphological changes in cells and the activation of specific caspase and mitochondrial regulatory pathways.^193–195^ The major apoptotic pathways are categorized into the mitochondrial pathway and death receptor pathways. Intracellular apoptotic signals typically activate the mitochondrial pathway,which stimulates the binding of BH3-only proteins with Bcl-2, leading to the aggregation of Bax/Bak on the mitochondrial membrane.^196^ This process triggers the release of pro-apoptotic proteins from the mitochondria, including cytochrome c and apoptosis-inducing factor (AIF), which subsequently activate caspases to initiate apoptosis. The extrinsic death receptor pathway begins with the binding of specific death receptors to ligands such as tumor necrosis factor-related TRAIL, TNF-α, and FASL, forming a death-inducing signaling complex that activates caspase-8, leading to apoptosis through a caspase cascade.^197^

**Fig. 4:**
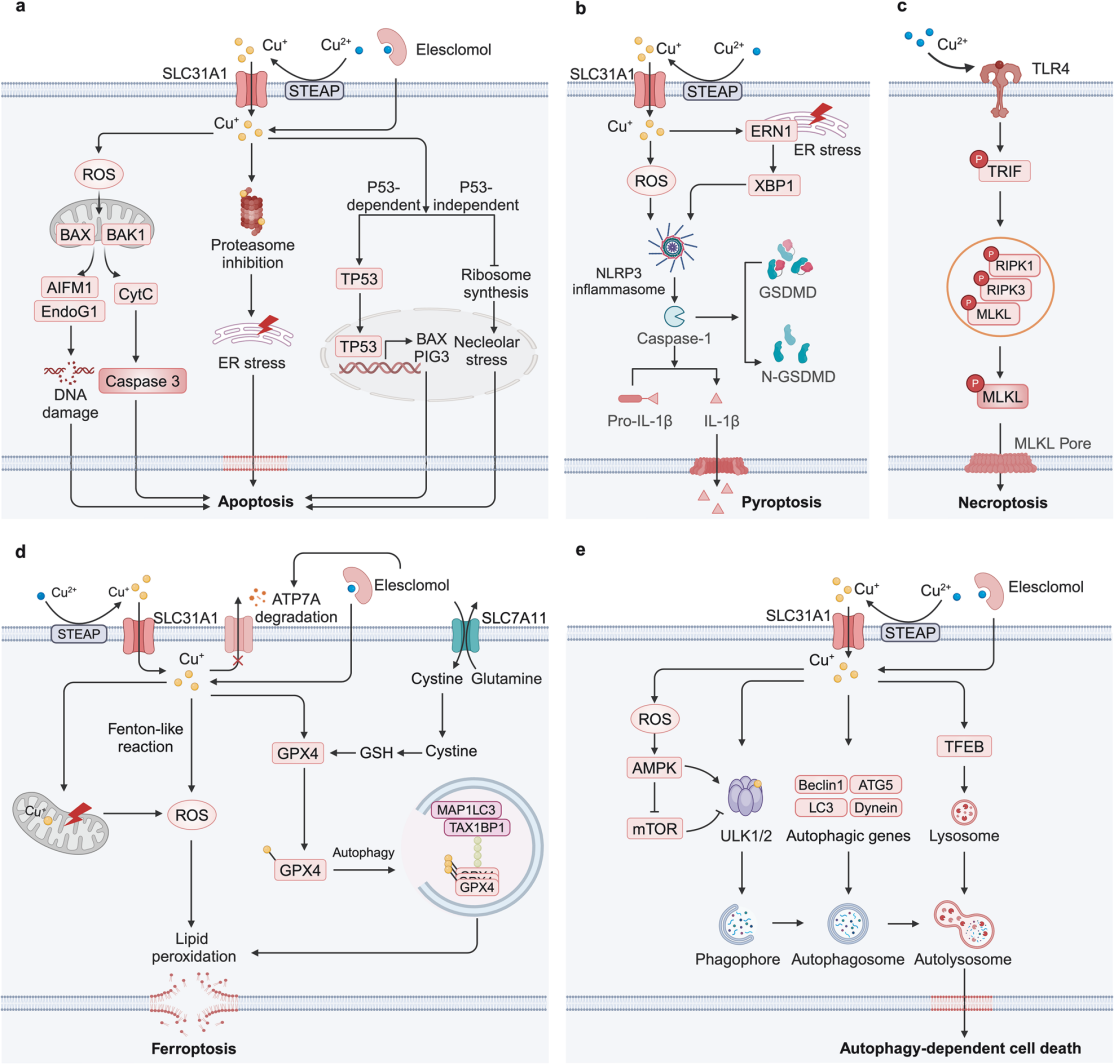
Mechanisms of copper-induced cell death. **a** Copper induces apoptosis primarily through the induction of ROS, DNA damage, and proteasome inhibition. Additionally, Copper induces TP53-dependent apoptosis by activating the transcription of TP53 target genes, while also triggering TP53-independent apoptosis through the inhibition of ribosomal synthesis and the induction of nucleolar stress. **b** Copper promotes pyroptosis by inducing ROS production and ER stress, resulting in NLRP3 inflammasome formation and membrane pore creation via GSDMD activation. **c** Copper toxicity activates the TLR4/NF-κB signaling pathway through oxidative stress, resulting in the phosphorylation and oligomerization of RIPK3 and MLKL, thereby triggering necroptosis. **d** Copper induces intracellular ROS through Fenton-like reactions and mitochondrial damage, leading to lipid peroxidation. It also binds to and induces the oligomerization of GPX4, promoting its autophagic degradation via the receptor TAXIBP1. **e** Copper initiates autophagy by activating AMPK, inhibiting mTOR, or directly binding to ULK1/2 kinases. Copper-mediated upregulation of autophagic genes and activation of the transcription factor TFEB contribute to the formation of autophagosomes and autolysosomes, which further promoting autophagy-dependent cell death. Created by BioRender. Abbreviations: ROS reactive oxygen species, ER endoplasmic reticulum, NLRP3 nod-like receptor family pyrin domain containing 3, GFDMD gasdermin D, TLR4 toll-like receptor 4, RIPK3 receptor-interacting protein kinase 3, MLKL mixed lineage kinase domain-like protein, GPX4 glutathione peroxidase 4, TAXIBP1 Tax1-binding protein 1, AMPK adenosine monophosphate-activated protein kinase, mTOR mammalian target of rapamycin, TFEB transcription factor EB

Excessive copper can induce apoptosis through multiple molecular mechanisms. Firstly, copper can catalyze the production of ROS when present in excess, leading to oxidative stress^198–200^ (Fig. 4a). The oxidative damage to cellular components such as lipids, proteins, and DNA can trigger the apoptotic pathways. Additionally, the accumulation of ROS can disrupt mitochondrial function, resulting in a loss of mitochondrial membrane potential and the release of pro-apoptotic factors like cytochrome c and AIF into the cytosol, which activates the caspase cascade and causes DNA fragmentation, leading to apoptosis.^201^ Secondly, copper can directly or indirectly influence the activity of various proteins involved in apoptotic pathways. For instance, copper binds to and activates proteins like p53, a key regulatory protein in apoptosis, thereby enhancing its ability to promote cell death in response to DNA damage. In human breast cancer MCF7 cells, copper-induced transactivation of P53 enhances the expression of BAX, a Bcl-2 family member, and the p53-induced gene 3 product, leading to the opening of mitochondrial permeability transition pores and the subsequent production of ROS.^202^ This research highlights the mechanism by which copper amplifies P53 transcriptional activity to promote apoptosis. Thirdly, endoplasmic reticulum (ER) stress and nucleolar stress are also significant contributing factors to cellular death under copper exposure. Copper sulfate-induced ER stress promotes apoptosis in mouse hepatocytes by activating the CHOP, JNK, and caspase-12 signaling pathways.^203^ Furthermore, copper-induced nucleolar stress impedes ribosomal synthesis and triggers a p53-independent apoptotic pathway.^204^ Notably, DSF also robustly inhibits the activity of the 26S proteasome in various cancer cell lines in a copper-dependent manner, ultimately leading to cancer cell apoptosis.^205^ Additionally, the activation of the TNF receptor-1 (TNF-R1) signaling pathway is involved in the copper-induced extrinsic apoptosis pathway, marked by substantial increases in the mRNA and protein levels of TNF-R1, Fas-associated death domain, TNFR-associated death domain, and cleaved caspase-8.^206^ These findings provide compelling evidence that copper can induce apoptosis via multiple molecular pathways, underscoring its potential utility as a targeted strategy in cancer treatment. It is also worth mentioning that research has demonstrated copper can induce apoptosis through a mechanism that does not involve caspases, although the exact processes remain unclear.^207^

### Copper and pyroptosis

Pyroptosis is a lytic form of PCD triggered by inflammasomes, primarily driven by the activation of caspase family proteins, including classical caspase-1 and non-classical caspase-4/11 or Caspase-5.^208–210^ This process is characterized by the cleavage of gasdermins (GSDMD) and the release of IL-1β and IL-18.^211^ The NLRP3 protein acts as a sensor for the mature inflammasome and is responsible for assembling the classical inflammasome during inflammatory stimulation.^212^

Copper-induced ROS generation and ER stress may contribute to pyroptosis (Fig. 4b). The accumulation of copper in hepatocytes leads to the expression of pyroptosis-related genes, including caspase-1, IL-18, IL-1β, and NLRP3.^213^ Likewise, exposing murine microglia to copper initiates an inflammatory response, activating the NLRP3/caspase-1/GSDMD axis and inducing cell pyroptosis.^214^ These effects are mediated by the activation of ROS/NF-κB pathway and subsequent disruption of mitophagy.^214^ Comparable outcomes have been observed in murine macrophages exposed to copper oxide nanoparticles, which induce oxidative stress and activate the NLRP3 inflammasome, leading to the expression of pro-IL-1β through the myeloid differentiation factor 88 (MyD88)-dependent TLR4 signaling pathway, subsequently activating NF-κB in macrophages.^215^ Besides, excessive copper increases the expression of pyroptosis-related genes in jejunal epithelial cells, primarily through the activation of the ER stress pathway.^216^ ER stress inhibitors, such as 4-phenylbutyric acid, can reduce copper-induced pyroptosis.^216^ These findings demonstrate there is crosstalk between copper and pyroptosis, necessitating further research to elucidate the precise underlying mechanisms and explore the implications of their interaction.

### Copper and necroptosis

Necroptosis is a form of programmed necrosis, mediated by the interaction of two receptor-interacting protein kinases (RIPK1 and RIPK3) and the mixed lineage kinase domain-like pseudokinase (MLKL).^217–219^ RIPK3 regulates the phosphorylation of MLKL, inducing its oligomerization and translocation to the plasma membrane, where it forms pore complexes and results in the release of DAMPs, cellular swelling, and membrane rupture.^220–222^

Emerging evidence indicates that copper and copper-based compounds play a role in the activation of necroptosis (Fig. 4c). Excess copper can activate genes associated with necroptosis, including RIPK1, RIPK3, and MLKL. Moreover, DNA damage caused by copper (II) can be alleviated by necroptosis inhibitors.^223,224^ Copper toxicity activates the Toll-like receptor 4 (TLR4)/NF-κB signaling pathway through oxidative stress, leading to the phosphorylation and oligomerization of RIPK3 and MLKL, thereby triggering necroptosis.^25^ These studies suggest a close relationship between copper and necroptosis; however, the direct mechanisms underlying this interaction remain to be elucidated.

### Copper and ferroptosis

Ferroptosis is an RCD driven by iron-dependent lipid peroxidation, primarily triggered by both intrinsic and extrinsic pathways.^225–227^ The extrinsic pathway inhibits the cystine/glutamate antiporter system XC^-^, which is essential for maintaining intracellular cystine and preventing lipid peroxide accumulation.^228^ The intrinsic pathway induces ferroptosis by inhibiting glutathione peroxidase 4 (GPX4), a critical enzyme that protects cells from oxidative stress.^229^

Both copper and iron possess potent redox potentials, enabling them to induce the production of hydroxyl radicals via Fenton or Fenton-like reactions^230,231^ (Fig. 4d). While previous research suggested that iron was the sole metal ion triggering ferroptosis,^232^ emerging evidence indicates that copper can also facilitate this form of cell death under certain conditions. Gao et al. have unveiled the potential mechanisms of anti-tumor mediated by the copper ionophore Elesclomol (ES)/Cu.^233^ Distinguished from other copper ionophores, ES exhibits the distinctive property of facilitating the degradation of ATP7A. Combining ES with copper leads to copper retention in mitochondria due to the degradation of ATP7A, which causes an accumulation of ROS. This promotes the degradation of SLC7A11, further intensifying oxidative stress and subsequent ferroptosis in CRC cells.^233^ Additionally, copper facilitates ferroptosis by inducing the autophagy of GPX4. Exogenous copper increases the ubiquitination of GPX4 through direct binding to the cysteine residues C107 and C148 of the GPX4 protein, promoting the formation of GPX4 aggregates. Tax1-binding protein 1 (TAX1BP1) serves as an autophagic receptor, orchestrating the degradation of GPX4, which collectively leads to ferroptosis in response to copper stress. The utilization of copper chelators can attenuate ferroptosis susceptibility without inhibiting other types of cell death.^22^ DSF/Cu is a promising anticancer drug with potential clinical applications. One mechanism by which it exerts its anticancer effects involves the mediation of ferroptosis through the activation of the ROS/MAPK and p53 signaling pathways.^234^ DSF/Cu treatment also profoundly impairs mitochondrial homeostasis, increasing the free iron pool and exacerbating lipid peroxidation, ultimately culminating in ferroptosis triggered by p62 phosphorylation-mediated NRF2 accumulation.^235^ Notably, a few studies have reported that copper may possess anti-ferroptotic properties, facilitated by a positive feedback loop mechanism between HIF1α and ceruloplasmin.^236^ Increased copper can also enhance the expression of GPX4, thereby impeding ferroptosis.^237^ These findings suggest that the complex interactions between copper and ferroptosis merit further investigation.

### Copper and autophagy

Autophagy, a universal cellular catabolic route, is orchestrated by autophagy-related proteins (ATGs) and associated factors.^238,239^ This dynamic recycling system facilitates the formation of membranous structures such as phagophores, autophagosomes, and autolysosomes.^240^ The initiation of autophagy is triggered by the modulation of kinase activity, primarily through the activation of AMPK or the inhibition of the mTOR pathway.^241,242^ As a pivotal energy monitor, AMPK facilitates autophagic processes by phosphorylating several targets including mTOR complex 1 (mTORC1), ULK1, and the BECN1 (beclin 1) component within the phosphatidylinositol 3-kinase catalytic subunit type 3 (PIK3C3/VPS34) complex.^243,244^ Conversely, active mTORC1 suppresses autophagy by engaging and phosphorylating the ULK ensemble, which comprises ULK1, ULK2, and ATG13.^245^ Autophagy plays a dual role in cellular processes, acting as both a suppressor and a promoter of cell death. Here, we will focus on the regulation of autophagy by copper and its facilitative role in cell death.

Copper can promote autophagy through multiple mechanisms (Fig. 4e). Firstly, copper activates the autophagy process by upregulating the expression of autophagy-related genes such as Beclin1, ATG5, LC3, mTOR, and Dynein,^246,247^ while also activating autophagic kinases ULK1 and ULK2.^128^ Secondly, intracellular copper overload induces autophagy via a ROS-dependent AMPK-mTOR pathway.^248^ Additionally, excessive copper increases cellular autophagy levels through the Cu-mTORC1-TFEB signaling pathway, where TFEB acts as a regulator of lysosomal biogenesis.^127^

Copper-induced autophagy can also facilitate cell death in cases of overactivation, particularly through ferroptosis, a form of autophagy-dependent cell death.^249^ Specifically, copper-induced production of ROS can activate autophagy pathways leading to the degradation of anti-ferroptotic factors such as SLC7A11, ferritin, lipid droplets, GPX4, and Cadherin 2.^250–253^ Additionally, copper has been shown to promote ferroptosis in pancreatic cancer cells by triggering TAX1BP1-mediated autophagic degradation of GPX4 protein.^22^ Moreover, various copper compounds, including copper oxide nanoparticles^254^ and copper (II) complexes,^255^ have been demonstrated to induce autophagic cell death in cancer cells, an effect that can be enhanced by autophagy inhibitors.

### Copper and the immune activation

Copper exerts tumor-suppressing functions by activating the immune cells. The cell membrane protein CD44, which serves as the primary receptor for hyaluronic acid and a regulator of cellular plasticity, can mediate an increase in intracellular copper. Within mitochondria, copper ions catalyze the oxidation of NADH molecules by hydrogen peroxide, resulting in the production of NAD^+^ ions. This biochemical process drives the basal metabolic activation of immune cells, specifically macrophages.^256^ DSF/Cu has demonstrated anti-tumor effects by provoking immunogenic cell death (ICD). ICD refers to a form of cell death that elicits an immune response against the antigens of dying cells. Cancer cells dying through ICD can release damage-associated molecular patterns (DAMPs) that are recognized by the immune system, thereby helping to prime the immune system against the tumor.^257^ Treatment with DSF/Cu enhanced the activation and maturation of dendritic cells (DCs). Furthermore, the additional blockade of CD47 further boosts DC maturation and enhances the cytotoxicity of CD8^+^ T cells. Mechanically, DSF/Cu facilitated the nuclear accumulation of Npl4, which disrupts the UPS and induces ER stress, leading to immune activation in HCC and improving the efficacy of CD 47 blockage.^258^ Moreover, macrophages can adopt different phenotypes within the tumor microenvironment, with M1 macrophages generally having pro-inflammatory and anticancer properties. DSF/Cu has been shown to promote the polarization of macrophages toward the M1 phenotype and to rewire glucose metabolism via the mTOR pathway. This shift supports antitumor immunity as M1 macrophages are efficient in phagocytosing tumor cells and presenting tumor antigens.^259^

### Cuproptosis in cancer

Based on the aforementioned studies, copper has been proven to induce various forms of cell death. However, emerging research indicates that copper-induced cell death represents a distinct form of cellular demise, which has been termed cuproptosis.

### Major milestone of cuproptosis

**Fig. 5:**
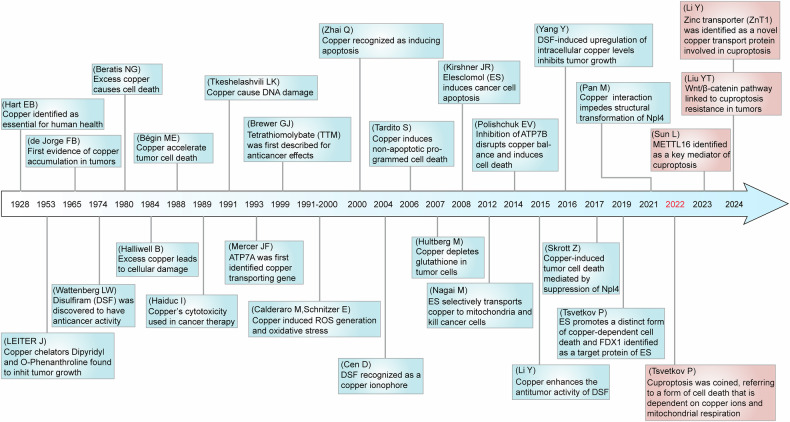
Major milestone of cuproptosis. The significance of copper ions has been recognized in 1928. Since 2022, research on cuproptosis and its regulatory mechanisms has surged. This timeline illustrates the evolution from the initial understanding of copper-induced cell death to the establishment of cuproptosis over the past few decades, providing insights into the major milestone surrounding cuproptosis and advancements in oncological research related to copper-associated cell death

Over the past few decades, the evolution from understanding copper-induced cell death to the discovery of cuproptosis has reflected researchers’ growing focus on this field (Fig. 5). The term “cuproptosis” was introduced in 2022 to describe a unique form of cell death characterized by copper accumulation and its distinct mechanisms.^26^ However, the journey leading to this discovery has roots in earlier observations regarding the critical biological roles of copper. As early as 1928, Hart et al. identified copper as an essential element for the production of red blood cells in rats fed a milk-based diet, thereby highlighting the necessity of copper for human health.^260^ In 1965, de Jorge and colleagues provided the first evidence linking tumors to accumulated copper by demonstrating an 11-fold increase in copper concentrations in brain cancer.^261^ From 1980 onward, researchers began to understand that excess copper could be toxic, potentially leading to cellular damage and even cell death.^262,263^ A further study in 1988 corroborated that copper could accelerate the death of tumor cells.^264^ Consequently, the cytotoxic properties of copper are increasingly being harnessed in anticancer therapies.^265,266^

During the 1990s, research increasingly focused on how copper induces cell damage through oxidative stress mechanisms. In 1991, copper was demonstrated to cause DNA damage by localizing on DNA and enhancing the production of oxygen radicals, particularly hydrogen peroxide (H_2_O_2_).^267^ However, whether there is an increased incidence of cancer in diseases associated with copper accumulation, such as WD and MD, remains to be determined.^267^ Then in 1993, the copper-transporting ATP7A was first isolated and identified among the candidate genes for MD.^268^ By 1994, research indicated that copper-induced ROS damage leads to DNA mutations, potentially serving as a mechanism for cancer development.^269^ Subsequent studies have also confirmed that superoxide anions and ROS, generated by copper within cells, can damage cellular lipids, proteins, and DNA.^270–272^ In 2000, Zhai et al. investigated the cytotoxic effects of copper and its underlying molecular mechanisms, revealing that copper (II) enhances the expression of Bax and ROS, subsequently inactivating NFκB and inducing apoptosis in the murine pre-B cell line BA/F3b.^273^ Until 2006, studies indicated that copper-induced human cell death could signify a unique form of non-apoptotic PCD, overturning prior constraints in our understanding and marking a milestone in the identification of “cuproptosis” as a novel mode of cell death.^274^ Additionally, it was reported that cell death induced by intracellular glutathione depletion depends on trace amounts of extracellular copper in 2007.^275^ In models related to WD, the disruption of copper balance through the suppression of ATP7B was also observed to induce cell death in 2014.^276^

Concurrently, the discovery and development of cuproptosis have been largely driven by explorations into the anticancer effects of copper-based compounds. By 1953, the discovery of copper chelators such as Dipyridyl and O-Phenanthroline, which could inhibit tumor growth, initiated the exploration of copper’s potential therapeutic applications.^277^ DSF, traditionally used as an alcohol abuse deterrent, was found to possess anticancer activity in 1974.^278^ However, it was not recognized as a copper ionophore until 2004 and is believed to cause cell death through mechanisms involving copper.^279^ The anticancer effects of TTM, a copper-chelating agent, were first described in 1999.^280^ A study conducted in 2000 revealed that copper-containing drugs could induce tumor cell death.^281^ Another copper ionophore, ES, demonstrated the capacity to induce cancer cell apoptosis in 2008.^282^ Copper ionophores are lipophilic molecules that reversibly bind copper and facilitate their transport across cellular membranes, including the plasma and mitochondrial membranes.^283^ While the exact mechanism of cell death induced by copper ionophores remains incompletely understood, research has suggested that it may involve the generation of ROS. Mitochondria, known for their role in cellular metabolism, are implicated in this process, as ROS are primarily derived from intracellular redox reactions, with mitochondria playing a significant role.^284^ Studies on cell death induced by ES indicated that elevated ROS levels, resulting from mitochondrial dysfunction contribute to cell death.^285–288^ In 2015, copper was reported to enhance the antitumor activity of DSF.^289^ Research in 2016 elucidated the anticancer mechanism of the DSF-copper complex, demonstrating that the DSF-dependent upregulation of intracellular copper concentration suppressed tumor growth through elevated ROS levels.^290^ In 2017, Npl4, an adaptor of the p97 segregase (also known as VCP), was identified as a molecular target for the tumor-suppressive effects of the DSF/Cu complex.^291^ Further research in 2021 revealed that copper induces the aggregation of p97-Npl4 by inhibiting ubiquitinated protein degradation, or directly binding to Npl4 and impeding its conformational changes, leading to cell death.^292^

The pioneering work of the team led by Peter Tsvetkov and Todd R. Golub established the foundation of cuproptosis. They discovered that ES can promote a distinct form of copper-dependent cell death and initially proposed the concept of cuproptosis.^293^ Notably, ferredoxin1 (FDX1) was identified as the direct target protein of ES, which interacts directly with ES-Copper and inhibits the formation of iron-sulfur clusters (Fe-S clusters).^293^ Additionally, subsequent research in 2021 on glioblastoma stem-like cells (GSCs) showed that ES/Cu directly targets the mitochondrial membrane, inducing a significant increase in mitochondrial ROS, ultimately leading to non-apoptotic, copper-dependent cell death.^294^ Until March 2022, Tsvetkov et al. formally defined this form of cell death as cuproptosis, which is distinct from known death mechanisms and reliant on copper and mitochondrial respiration, marking a significant advancement in the field.^26^ In the subsequent years, research primarily focused on further elucidating the mechanisms of cuproptosis and exploring anticancer drugs based on cuproptosis. In 2023, Sun et al. identified the atypical methyltransferase METTL16 as a key mediator of cuproptosis through its m6A modification of FDX1 mRNA.^295^ They established the copper-lactylated METTL16-FDX1-cuproptosis axis as a crucial regulatory mechanism in copper-related metabolism, filling a significant gap in our understanding of cuproptosis regulation. Liu et al. uncovered the abnormal activation of the Wnt/β-catenin signaling pathway imparts resistance to cuproptosis in tumor cells, proposing a precision medicine strategy for cancer treatment through the selective induction of cuproptosis.^296^ In the same year, Li et al. demonstrated that zinc transporter 1 (ZnT1) is a novel copper transport protein capable of mediating copper (II) uptake and inducing cuproptosis.^297^ Collectively, these studies contribute to a deeper understanding of the mechanisms and regulation of cuproptosis, highlighting its significance for therapeutic interventions.

## Molecular mechanism of cuproptosis

**Fig. 6:**
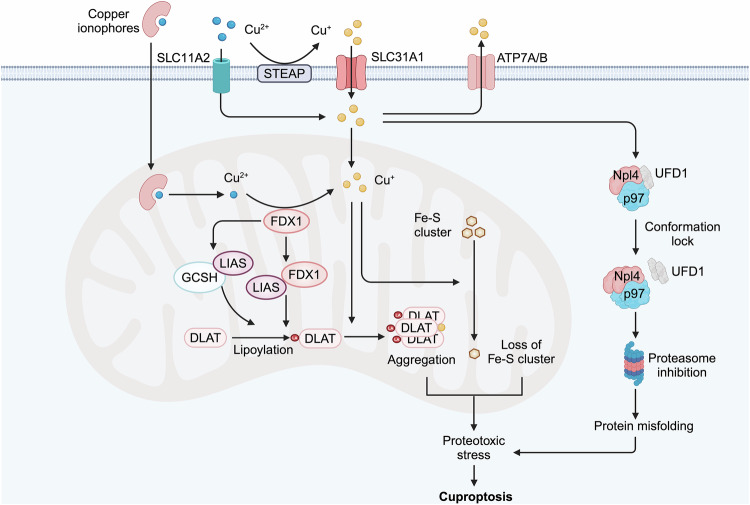
Mechanism of cuproptosis. The mechanism of cuproptosis includes both mitochondrial-dependent and mitochondrial-independent pathways. Excess copper (II) enters the mitochondria, where it is reduced to the more toxic copper (I) by the mitochondrial protein FDX1. FDX1 also promotes protein lipoylation by directly binding to the LIAS and enhancing its interaction with the GCSH. Copper (I) binding induces the aggregation of DLAT and destabilizes Fe-S cluster proteins, triggering cellular stress responses that result in cuproptosis. Additionally, DSF/Cu mediates the aggregation and conformation lock of the Npl4-p97 protein in the cytoplasm, inhibiting the ubiquitin-proteasome degradation pathway, which contributes to proteotoxic stress and cuproptosis. Created by BioRender. Abbreviations: FDX1 ferredoxin 1, LIAS lipoic acid synthase, GCSH glycine cleavage system protein H, DLAT Dihydrolipoamide S-Acetyltransferase, Fe-S cluster iron-sulfur cluster, DSF Disulfiram, Npl4 an adaptor of the p97 segregase (also known as VCP)

Cuproptosis is a novel type of regulated cell death that depends on mitochondrial metabolism.^26,293^ However, recent research has unveiled the existence of mitochondrial-independent cuproptosis. Here, we delineate its core mechanisms, specifically emphasizing the mitochondrial-dependent and mitochondrial-independent pathways (Fig. 6).

### Mitochondria-dependent cuproptosis

Cuproptosis is different from the current well-known cell death, because using the inhibitors or knockouting these pathway genes failed to rescue cuproptic cells. However, cancer cells with mitochondrial respiration are significantly more sensitive to ES than those with glycolysis, suggesting the pivotal role of mitochondria in cuproptosis. Further studies using whole-genome CRISPR-Cas9 knockout screening identified FDX1, LIAS, and DLAT, which are components of the lipoic acid (LA) pathway or the pyruvate dehydrogenase complex, as essential genes required for cuproptosis.^26,293^ Excess copper (II) enters the mitochondria, where it is reduced to the more toxic copper (I) by the mitochondrial protein FDX1. FDX1 also promotes protein lipoylation by directly binding to lipoic acid synthase (LIAS) and enhancing its interaction with the glycine cleavage system protein H (GCSH).^298^ Notably, mitochondrial copper can directly bind to proteins following their lipoylation, a post-translational modification involving the covalent attachment of an eight-carbon organosulfur lipoic acid moiety to specific lysine residues, leading to the aggregation of lipoylated proteins.^299,300^ For example, copper binding induces the aggregation of dihydrolipoamide S-acetyltransferase (DLAT).^301^ Moreover, mitochondrial copper destabilizes Fe-S cluster proteins, which are crucial for protein lipoylation and electron transfer reactions in mitochondria. Consequently, copper toxicity results in improper folding of DLAT and the loss of proteins containing Fe-S clusters, leading to a cascade of cellular stress responses characterized by the increase of heat shock proteins, that ultimately culminate in cuproptosis.

### Mitochondria-independent cuproptosis

FDX1 is a core regulator of mitochondria-dependent cuproptosis, as mentioned above. However, Attar et al. identified histone H3-H4 tetramer as a novel copper (II) reductase in the eukaryotic cells, indicating the fungibility of FDX1 in cuproptosis.^302^ Gale et al. demonstrated that ES/Cu induces FDX1-independent astrocyte toxicity mediated by oxidative stress, as FDX1 knockdown did not block ES/Cu toxicity to astrocytes.^303^ Additionally, inhibition of mitochondrial respiration failed to rescue the ES/Cu toxicity,^303^ and intracellular copper can be released from the ES/Cu complex and become bioavailable outside the mitochondria, suggesting a great likelihood of mitochondrial-independent cuproptosis.^304^ Similarly, DSF/Cu can mediate the aggregation and lock the conformational transition of cytoplasmic p97 complex that plays a central role in cellular protein homeostasis, inhibiting cellular ubiquitin-proteasome degradation pathways and further leads to increased proteotoxic stress and cell death.^291,292,305^ These findings highlight the existence of mitochondria-independent cuproptosis, although the molecular mechanisms remain inadequately understood. Hence, further exploration of mitochondrial-independent cuproptosis could trigger a novel breakthrough in the field of cuproptosis.

### Functions of cuproptosis in cancer biology

Cuproptosis has emerged as a significant player in cancer biology. On one hand, it appears to function as an innate mechanism for tumor suppression. On the other hand, cancer cells evade cuproptosis, thereby promoting tumor progression and treatment resistance.

### Cuproptosis induction in tumor suppression

**Fig. 7:**
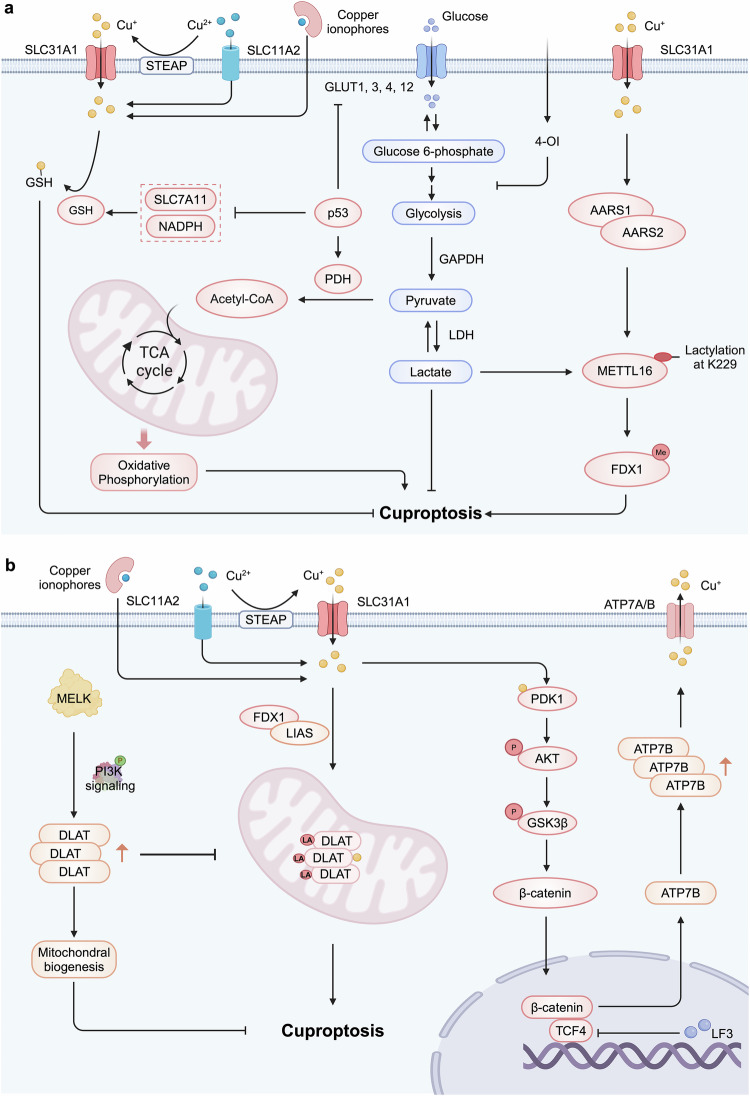
Cancer-related pathways in cuproptosis. **a** Cuproptosis induction in tumor suppression. The tumor suppressor p53 inhibits glucose uptake and glycolysis, shifting metabolism towards the TCA cycle and oxidative phosphorylation, which sensitizes cells to cuproptosis. It also suppresses NADPH production by inhibiting G6PD and the pentose phosphate pathway, resulting in decreased GSH levels. Intratumoral copper enhances METTL16-K229 lactylation and activity through its interaction with AARS1 or AARS2, leading to increased FDX1 expression and cuproptosis induction. Additionally, the metabolite 4-OI alkylates cysteine residues in GAPDH, inhibiting its activity and suppressing aerobic glycolysis, thereby further promoting cuproptosis. **b** Cuproptosis evasion in tumor progression and therapeutic resistance. Increased MELK expression in tumors activates the PI3K/mTOR signaling pathway, leading to elevated DLAT expression and stabilized mitochondrial function, which enhances resistance to cuproptosis. Additionally, copper binds to PDK1, activating the downstream AKT-GSK3β-β-catenin pathway and promoting CSC characteristics. CSCs demonstrate heightened resistance to cuproptosis, as the β-catenin/TCF4 transcription complex binds to the ATP7B promoter, facilitating copper expulsion. Created by BioRender. Abbreviations: TCA tricarboxylic acid cycle, NADPH nicotinamide adenine dinucleotide phosphate, G6PD glucose-6-phosphate dehydrogenase, GSH glutathione, METTL16 methyltransferase-like 16, 4-OI 4-octyl itaconate, GAPDH glyceraldehyde-3-phosphate dehydrogenase, MELK maternal embryonic leucine zipper kinase. CSC cancer stem cell

The tumor suppressor protein p53 inhibits tumor development partly by inducing cuproptosis (Fig. 7a). P53 serves as a crucial metabolic regulator in the modulation of glycolysis and oxidative phosphorylation, two tightly coupled metabolic processes closely linked to cellular sensitivity to cuproptosis.^306–308^ Specifically, p53 inhibits glucose uptake and glycolysis, promoting a metabolic shift towards the tricarboxylic acid (TCA) cycle and oxidative phosphorylation, thereby increasing sensitivity to cuproptosis.^309^ Additionally, p53 regulates the biosynthesis of GSH to facilitate cuproptosis. Mechanistically, p53 suppresses the production of NADPH by inhibiting malic enzymes and G6PD, as well as the associated pentose phosphate pathway.^310,311^ NADPH is a crucial reductant for the regeneration of GSH, and its reduction leads to decreased GSH levels. Consequently, p53-mediated metabolic remodeling and cuproptosis may represent an effective strategy for eradicating cancer cells.

The epigenetic regulator METTL16 plays a vital role in tumor suppression by promoting cuproptosis. In mechanism, intratumoral copper ions promote the lactylation and activity of METTL16-K229 by increasing its interaction with AARS1 or AARS2. Lactylation of METTL16 induces methylation modifications at the FDX1-602 site, thus enhancing the expression of FDX1 and leading to cuproptosis. In contrast, SIRT2, a classical deacetylase, significantly inhibits the lactylation of METTL16 and the m6A modification of FDX1, thereby impeding cuproptosis.^295^ Consistently, AGK2, a SIRT2-specific inhibitor, promotes the therapeutic effects of ES by inducing the lactylation of METTL16-mediated cuproptosis in gastric tumors in vitro and in vivo. These findings suggested that the lactylation modification of METTL16 inhibits the development of gastric cancer partly through the induction of cuproptosis.

Tumor metabolites also inhibit tumor progression by inducing cuproptosis. For example, 4-octyl itaconate (4-OI), a derivative of the TCA cycle metabolite produced by aconitate decarboxylase 1, has been shown to promote cuproptosis by inhibiting aerobic glycolysis.^312^ Specifically, 4-OI alkylates the cysteine residues of GAPDH, inhibiting the enzymatic activity and thereby leading to the suppression of aerobic glycolysis. Meanwhile, another metabolite derived from the TCA cycle intermediate fumarate, dimethyl fumarate, can also inhibit aerobic glycolysis by targeting GAPDH, potentially promoting cuproptosis.^313^

### Cuproptosis evasion in tumor progression and therapeutic resistance

Despite the presence of a cuproptosis-mediated tumor suppression mechanism, tumors can still arise and progress uncontrollably, suggesting the existence of evasion mechanisms against cuproptosis in cancer cells (Fig. 7b). The oncogene maternal embryonic leucine zipper kinase (MELK) functions as a suppressor of cuproptosis to contribute to tumorigenesis and tumor progression. Elevated expression of MELK in tumors enhances the activity of the PI3K/mTOR signaling pathway, which subsequently boosts the expression of DLAT and stabilizes mitochondrial functions.^314^ The increase in DLAT (not the oligomer, possibly lipoic acid-modified DLAT) helps improve mitochondrial respiration, eliminates excessive intracellular ROS, and also promotes resistance to ES-induced cuproptosis, thus contributing to the tumorigenesis and progression of HCC.

Additionally, aberrant activation of the Wnt/β-catenin signaling pathway endows tumor cells with the ability of therapeutic resistance by evading cuproptosis. Liu et al. indicated that the process of cuproptosis is accompanied by robust activation of the Wnt/β-catenin pathway.^296^ Mechanistically, copper ions in tumors directly bind to PDK1, activating the downstream AKT-GSK3β-β-catenin pathway and enhancing cancer stem cells (CSCs) traits. Interestingly, CSCs exhibit greater resistance to cuproptosis because the β-catenin/TCF4 transcriptional complex can directly bind to the ATP7B promoter and induce its expression, which is responsible for reducing intracellular copper levels. CSCs are often characterized by multi-drug resistance within tumors.^315,316^ Therefore, aberrant activation of the Wnt/β-catenin pathway aids CSCs in adapting to therapeutic interventions by evading cuproptosis.

### Cuproptosis-mediated crosstalk within the tumor microenvironment (TME)

The TME refers to the multifaceted ecosystem surrounding tumor cells, encompassing various cellular and non-cellular components.^317,318^ These components interact intricately, significantly influencing tumor growth and progression.^319^ Tumor cells undergoing cuproptosis exhibit immunostimulatory effects in both direct and indirect manners. On one hand, cuproptotic tumor cells can directly activate their internal cyclic GMP-AMP synthase (cGAS)/stimulator of interferon genes (STING) signaling pathway, triggering the release of inflammatory factors to initiate immune responses within the TME. Specifically, cuproptosis-induced mitochondrial proteotoxic stress promotes the release of mitochondrial DNA (mtDNA), which acts as an intracellular DAMP,^320^ activating the tumor-immune-related mtDNA-cGAS-STING signaling pathway and leading to the secretion IFN-β and CXCL10.^321^ Subsequently, these cytokines facilitate DC maturation, cytotoxic CD8^+^ T cell infiltration, and natural killer (NK) cell recruitment, collectively promoting tumor regression in preclinical models. On the other hand, tumor cells undergoing curptoptosis release certain “eat me” signals, indirectly initiating a sustained anti-tumor immune response. The membranes of tumor cells damaged by cuproptosis release various DAMPs, such as ATP, HMGB1, and calreticulin (CRT), which enhance the maturation of DCs and the activation of CD8^+^ effector T cells, thereby triggering classical ICD.^322–328^ Intriguingly, the cGAS-STING signaling pathway is also activated in DCs by cuproptotic cancer cells, induced by ES and CuCl_2_, which subsequently enhances the release of inflammatory mediators including IL-2, TNF-α, IFN-γ, CXCL10, and CXCL11.^329^ These cuproptosis-mediated immunostimulatory effects were also observed in copper-based nanomedicine.^325,330–338^ For instance, TPP-CuET, a mitochondria-targeted copper complex modified with triphenylphosphine, effectively inhibits the mitochondrial tricarboxylic acid cycle, ATP synthesis, and the electron transport chain.^330^ Simultaneously, it activates the immune response of CD8^+^ T cells and NK cells via the MHC I pathway, enhancing antigen processing and presentation in cancer cells.^330^ A novel nucleic acid nanoplatforms can inhibit HIF-1 expression, thereby alleviating the immunosuppressive TME, and enhancing antigen presentation through the activation of Toll-like receptor 9 (TLR9) via the immune adjuvant polyCpG.^331^

However, cuproptosis also leads to the upregulation of PD-L1 protein in tumor cells, inhibiting the cytotoxic CD8^+^ T cell response.^339^ Consistently, elevated copper levels can promote PD-L1 expression by upregulating the JAK/STAT signaling pathway, thereby enhancing the negative regulatory effect on T cells that facilitates tumor immune escape.^20,189^ Moreover, certain nanomaterials such as CAT-ecSNA-Cu and NP@ESCu induce cuproptosis in tumor cells and increase PD-L1 expression, though creating favorable conditions for combined anti-tumor therapy with immunotherapy.^326,331^ These findings support the immunosuppressive effects of cuproptotic tumor cells, suggesting the complex immunoregulatory nature of cuproptosis.

### Clinical associations of cuproptosis-related genes (CRGs)

Tsvetkov et al. identified ten genes through whole genome knockout screening that may modify susceptibility to cuproptosis, classifying FDX1, LIAS, LIPT1, DLD, DLAT, PDHA1and PDHB as positive regulators, and MTF1, GLS and CDKN2A as negative regulators.^26^ Additional analysis on copper homeostasis indicated that SLC31A1, ATP7A, and ATP7B could also influence cuproptosis by regulating intracellular copper concentrations. Among these 13 CRGs, FDX1, DLAT, LIAS, SLC31A1, ATP7A, and ATP7B are well-studied, and therefore, we will explore their expression levels and clinical relevance across different cancers.

FDX1 is predominantly downregulated in various types of cancer tissues, particularly in solid tumors, including clear cell renal cell carcinoma (ccRCC), breast invasive carcinoma, colon adenocarcinoma, lung adenocarcinoma, thyroid carcinoma, and HCC. whereas, it is upregulated in certain female reproductive tumors such as ovarian serous cystadenocarcinoma (OV) and uterine corpus endometrial carcinosarcoma, as well as in glioblastoma (GBM).^340,341^ High levels of FDX1 correlate with poor prognosis in brain lower-grade glioma, while better outcomes are observed in ccRCC and HCC.^342–344^ Particularly in ccRCC, low expression of FDX1 is significantly associated with advanced TNM staging, lymph node metastasis, and poorer prognosis.^342^ Single-cell RNA sequencing analysis also indicates that FDX1 is expressed in immune cells, with notable variations in expression levels among monocytes or macrophages.^343^ The effect of FDX1-dependent cuproptosis in these immune cells needs further clarification.

DLAT, the E2 subunit of the PDCs, is critical for TCA cycle.^345,346^ In gastric cancer cells, DLAT expression is notably upregulated, enhancing oxidative phosphorylation by catalyzing the conversion of pyruvate to acetyl-CoA, thereby supplying energy to tumor cells.^347^ Studies in non-small cell lung cancer (NSCLC) have shown that PM2.5 upregulates DLAT expression through a dual-regulatory mechanism involving the Sp1-DLAT and eIF4E-DLAT axes, promoting glycolysis and enhancing tumor cell proliferation.^348^ Based on multi-database and experimental verification, DLAT is significantly upregulated in ccRCC^349^ and HCC,^350^ revealing its role as a tumor suppressor gene.^351^ Guo et al. first explored the correlation between disease-free survival in bladder cancer patients and the expression of lipoylated DLAT protein targets using Gene Expression Profiling Interactive Analysis from the TCGA database.^326^ The findings indicate that patients with low DLAT expression have longer disease-free survival than those with high DLAT expression. Furthermore, DLAT expression positively correlates with the expression of FDX1 and LIAS. In bladder tumor tissues, DLAT expression is significantly positively correlated with PD-L1 expression. Additionally, the level of DLAT expression is positively associated with the immune infiltration levels of B cells, macrophages, neutrophils, and DCs.^351^

LIAS is linked to mitochondrial energy metabolism and antioxidant defense, functioning in the electron transport chain and oxidative phosphorylation within the TCA cycle. Therefore, tumors characterized by high levels of oxidative phosphorylation, such as breast cancer (BRCA), melanoma, and cholangiocarcinoma, are predominantly dependent on LIAS.^352^ Recent pan-cancer bioinformatics analyses have shown that high expression of LIAS correlates with favorable prognosis in patients with ccRCC, rectal adenocarcinoma, BRCA, and ovarian cancer. In contrast, high expression of LIAS is associated with poor prognosis in lung cancer patients.^353^ Furthermore, LIAS shows differential expression between recurrent and non-recurrent colorectal cancer samples.^354^

Dysregulation of copper homeostasis, mediated by factors such as SLC31A1, ATP7A, and ATP7B, can lead to cellular dysfunction.^4,26^ As previously mentioned, SLC31A1 mediates copper entry into cells, while ATP7A and ATP7B facilitate copper efflux, collectively functioning as copper carriers intimately involved in copper shuttling.^355–358^ In research focused on gliomas, SLC31A1 was considered a risk factor, whereas ATP7B was deemed a protective factor, further suggesting a potential connection between copper homeostasis and cancer.^359^ Furthermore, studies have established that microsatellites of SLC31A1 and ATP7B are linked to an increased risk of lung cancer, implying that the expression levels of copper homeostasis-related genes could influence cancer progression.^360^ Two independent studies suggested that high expression of SLC31A1 correlates with adverse clinical outcomes in BRCA patients.^361,362^ Additionally, elevated levels of ATP7A are associated with poor overall survival (OS) in BRCA and liver cancer patients.^363,364^ Notably, SLC31A1 demonstrates a strong correlation with the TME. Upregulation of SLC31A1 is associated with poor OS in cervical squamous cell carcinoma, esophageal cancer, BRCA, or head and neck squamous cell carcinoma patients.^365^ The analysis of tumor immune infiltration revealed associations between the expression of SLC31A1 and the presence of T cells, and macrophages within the TME. Interestingly, the expression of SLC31A1 is negatively correlated with OS and tumor infiltration of plasmacytoid DCs, NK cells, and CD8^+^ T cells, but it is positively correlated with the high abundance of characteristic immunosuppressive immune cell types in tumors of glioma patients.^366^ In BRCA patients, tumor expression of SLC31A1 is positively correlated with the infiltration of immune cells including CD4^+^ T cells, macrophages, neutrophils, and DCs, as well as with the expression of immune checkpoint genes such as CD274 and CTLA4, despite its association with poorer prognosis.^366^ Finally, a study employing TCGA and tissue microarray data revealed a significant positive correlation between the expression of SLC31A1 and CD274 across multiple cancer types.^20^

DLD, PDHA1, and PDHB, as crucial subunits of the PDC, play significant roles in the process of cuproptosis.^345,367^ According to a study by Ma et al., the expression levels of DLD, PDHA1, and PDHB1 are notably higher than those of other CRGs in almost all tumors according to the TCGA database.^368^ Furthermore, the expression levels of these genes are also considered to be associated with tumor prognosis. For instance, high levels of PDHA1 are linked to better prognosis in lung cancer patients and may serve as a biomarker for immunotherapy response.^369^ Additionally, CDKN2A, GLS, and MTF1 have been shown to correlate with cellular sensitivity to cuproptosis.^26^ CDKN2A is often overexpressed in most cancer patients and is associated with adverse prognostic outcomes.^370^ In HCC, CDKN2A promotes tumor cell proliferation and migration capabilities as well as attenuating cuproptosis.^371^ Furthermore, CDKN2A serves as a target for puromycin, which may contribute to preventing cancer progression.^372^ GLS and MTF1 potentially influence cellular sensitivity to cuproptosis by regulating the levels of intracellular copper-binding substances, such as MTs and GSH.^373,374^ Notably, the mitochondrial copper (I) transporter PiC2 is a target of MTF1.^375^ The LIPT1 enzyme facilitates the activation of 2-oxoglutarate dehydrogenases within the TCA cycle.^376^ Inhibition of LIPT1 expression has been demonstrated to hinder the growth, invasion, and metastasis of HCC cells.^377^ Interestingly, LIPT1 also exhibits a positive correlation with PD-L1 in a variety of cancer types, further emphasizing its potential relevance in cancer biology.^378^

### Copper and cuproptosis targeting strategies for therapy

Copper and cuproptosis are intricately linked to cancer. Cellular copper levels are meticulously regulated, and its dyshomeostasis significantly inhibits tumor progression. Therefore, depleting copper and inducing cuproptosis through copper introduction presents new therapeutic strategies for cancer treatment. Notably, certain cancers characterized by enhanced mitochondrial respiration, stem cell-like traits, or drug resistance demonstrate greater responsiveness to cuproptosis-based therapies,^80^ indicating its potential as a valuable supplement to current chemotherapy, radiotherapy, and immunotherapy. Furthermore, cuproptosis-based therapies can enhance the sensitivity of cancer cells to these traditional treatments,^293,296,324,327,334,379–388^ making cuproptosis-based combination therapies a promising avenue for cancer treatment. Given these points, it is essential to deepen our understanding of copper chelators (Table 1) and ionophores (Table 2).

**Table 1:** Copper chelators in clinical trials for cancer therapy

Agent	Mechanism involved	Preclinical cancer type	Clinical cancer type	Combination	NCT	Phase
Tetrathiomolybdate (TTM)	Inhibits angiogenesis and metastasis	Ovarian carcinoma,^508^ Cervical cancer,^509^ Prostate cancer,^510^ Melanoma,^392^ Lung cancer,^407^ Pancreatic cancer,^356^ and Papillary thyroid cancer^394^	Hormone-refractory prostate cancer^408^	N/A	NCT00150995	II
			Hepatocellular carcinoma	N/A	NCT00006332	II
			Esophageal carcinoma^511^	N/A	NCT00176800	II
			Breast cancer^512^	N/A	NCT00195091	II
			Advanced kidney cancer^406^	N/A	N/A	II
			Metastatic non-small-cell lung cancer	Carboplatin and Pemetrexed	NCT01837329	I
			High risk for relapse of triple-negative breast cancer	Capecitabine and pembrolizumab	NCT06134375	Ib and II
ATN-224	Inhibits SOD1 activity	Metastatic head and neck squamous cell carcinoma,^410^ lung cancer,^513^ leukemia,^514^ diffuse large B cell lymphoma,^515^ and breast cancer^434^	Advanced solid tumors(breast, melanoma, colon,renal and others)^411^	N/A	N/A	I
			Advanced melanoma	Temozolomide	NCT00383851	II
			Prostate cancer	N/A	NCT00405574	II
			Multiple myeloma	Bortezomib	NCT00352742	I and II
			Biochemically-recurrent hormone-naive prostate cancer^412^	N/A	N/A	II
D-penicillamine (D-pen or PCA),	Inhibits angiogenesis and metastasis	Gliosarcoma,^516^ lung cancer,^417^ breast cancer,^417^ cervical cancer,^415^ and leukemia^517^	Recurrent head and neck cancer	N/A	NCT06103617	II
			Glioblastoma^418^	N/A	NCT00003751	II
Triethylenetetramine (TETA/trientine)	Inhibits angiogenesis and enhances apoptosis	Hepatocellular carcinoma,^422,423^ and mesothelioma^518^	Advanced malignancies^426^	Carboplatin	NCT01178112	I
			BRAF mutated metastatic melanoma	Vemurafenib	NCT02068079	I
			Epithelial ovarian cancer^425^	Pegylated liposomal doxorubicin and carboplatin	NCT03480750	I and II
Tetraethylenepentamine pentahydrochloride (TEPA)	Inhibits PD-L1 expression	Neuroblastoma^20^	N/A	N/A	N/A	N/A

**Table 2:** Copper ionophores in clinical trials for cancer therapy

Agent	Mechanism involved	Preclinical cancer type	Clinical cancer type	Combination	NCT	Phase
Elesclomol (ES)	Induces apoptosis, cuproptosis and ferroptosis	Breast cancer,^519^ colorectal cancer,^233,312^ thyroid cancer,^520^ uveal melanoma,^521^ gastric cancer,^295^ and prostate cancer^522^	Metastatic melanoma^441^	Paclitaxel	NCT00084214	I and II
			Chemotherapy-naïve with advanced melanoma^446^	Paclitaxel	NCT00522834	III
			Relapsed or refractory acute myeloid leukemia^445^	N/A	NCT01280786	I
			Solid tumors	N/A	NCT00827203	I
			Refractory solid tumors^444^	Paclitaxel	NCT00088114	I
			Metastatic prostate cancer	Docetaxel and prednisone	NCT00808418	I
			Recurrent ovarian epithelial cancer^443^	Paclitaxel	NCT00888615	II
			Soft-tissue sarcomas	Paclitaxel	NCT00087997	II
			Non-small-cell lung cancer	Paclitaxel and Carboplatin	NCT00088088	I and II
Disulfiram (DSF)	Induces apoptosis, cuproptosis and ferroptosis	Breast cancer,^291,523–525^ cervical cancer,^526^ chondrosarcoma,^452^ thyroid cancer,^527,528^ lung cancer,^524^ pancreatic cancer,^529^ hepatocellular carcinoma,^192,235,530^ and colon cancer^531^	Advance gastric cancer	Cisplatin	NCT05667415	N/A
			Recurrent glioblastoma^459^	Dietary copper and alkylating agents	NCT02678975	II and III
			Recurrent glioblastoma^532^	Copper gluconate and temozolomide	NCT03034135	II
			Newly diagnosed glioblastoma multiform	Temozolomide	NCT01777919	II
			Newly diagnosed glioblastoma^533^	Standard radiation therapy and temozolomide	NCT02715609	I and II
			Refractory germ cell tumors^457^	Cisplatin	NCT03950830	II
			Metastatic melanoma	N/A	NCT00256230	I and II
			Metastatic pancreatic cancer	Chemotherapy	NCT02671890	I
			Metastatic breast cancer	N/A	NCT03323346	II
			Treatment-refractofy sarcomas	Copper gluconate and liposomal doxorubicin	NCT05210374	I
			Refractory solid tumors involving the liver^458^	Copper gluconate	NCT00742911	I
			Treatment-refractory multiple myeloma	Copper gluconate	NCT04521335	I
			Metastatic castration-resistant prostate cancer^534^	Copper gluconate	NCT02963051	I
			Non-small-cell-lung cancer^456^	Chemotherapy	NCT00312819	II and III
Clioquinol (CQ)	Induces apoptosis and inhibits proteasome	Prostate cancer,^207,461,535,536^ breast cancer,^537,538^ ovarian cancer,^539^ and cervical cancer^540^	Advanced hematologic malignancies^541^	N/A	NCT00963495	I
8-hydroxyquinoline (8-OHQ)	Induces apoptosis and paraptosis, as well as inhibits proteasome	Breast cancer,^460^ lung cancer,^542^	N/A	N/A	N/A	N/A
NSC319726	Induces oxidative stress and cell-cycle arrest	Glioblastomas,^113^ chromophobe renal cell carcinoma^543^	N/A	N/A	N/A	N/A
Pyrithione	Induces ROS production and apoptosis	Breast cancer, hepatocellular carcinoma, and myeloma^544^	N/A	N/A	N/A	N/A

### Copper chelators

Copper chelators, which bind to copper and reduce its bioavailability, are a critical strategy for targeting copper homeostasis in cancer therapy. Various copper chelators have proven potential in preclinical animal research and clinical trials aimed at cancer treatment, primarily by inhibiting angiogenesis and impairing tumor cell proliferation and metastasis.^389,390^ Representative agents include TTM, choline tetrathiomolybdate (ATN-224), D-penicillamine (D-pen or PCA), Triethylenetetramine (TETA, commonly known as trientine), and Tetraethylenepentamine pentahydrochloride (TEPA).^390^

TTM is an oral copper chelator that has been shown to inhibit tumor angiogenesis and metastasis.^155,166,181,391^ Recent studies indicate that TTM further inhibits the activity of MEK1/2 kinases by lowering copper levels, contributing to the suppression of papillary thyroid cancer and colon cancer, as well as BRAFV600E-driven tumorigenesis in melanoma.^120,122,392–395^ Importantly, TTM enhances the antitumor efficacy of MEK1/2 and BRAFV600E inhibitors, including sorafenib and vemurafenib.^394^ In clinical trials with TTM, a Phase I study with advanced cancer patients found that most achieved copper deficiency after 6–8 weeks of treatment.^280^ The notable toxicity associated with TTM was anemia, defined as a hematocrit below 80% of baseline, occurring in about one-third of patients. Rapidly reversible neutropenia without infection was also observed. Anemia was considered directly related to the degree of copper deficiency rather than TTM dosage, and all patients recovered without the need for transfusions within 5–7 days after discontinuing TTM.^280^ In a study involving high-risk breast cancer patients in stages II-IV, 75 individuals received TTM as adjuvant treatment across two cycles (induction and maintenance).^396^ With a median follow-up of 6.3 years, the event-free survival rate for the entire cohort was 72%, and the OS rate was 84%. Among patients with stage II-III disease, the 2-year OS rate was 96%, while it was 93% for those with stage IV. TTM was well tolerated, with only 5.7% of patients experiencing grade 3 or 4 adverse events, primarily neutropenia, all of which were reversible. Furthermore, lower levels of ceruloplasmin were associated with a reduction in circulating angiogenic progenitor cells (VEGFR2+) and decreased serum LOXL-2 levels, indicating that TTM treatment may influence the premetastatic niche.^396^ Another study involving 30 patients with malignant pleural mesothelioma revealed that postoperative TTM treatment delayed disease progression in stage I and II patients, extending the progression-free survival (PFS) from 10 to 20 months.^397^ Compared to traditional multimodal therapies, TTM showed anti-angiogenic effects after tumor resection, with minimal toxicity and comparable efficacy.^397^ TTM also exhibits radiosensitizing and chemosensitizing effects,^398,399^ enhancing the efficacy of chemotherapeutic agents like doxorubicin, mitomycin C, fenretinide, 5-fluorouracil, and cisplatin in preclinical models without increasing side effects.^400–403^ A pilot trial of TTM in combination with irinotecan, 5-fluorouracil, and leucovorin for metastatic colorectal cancer also demonstrates that TTM can be safely incorporated into combination chemotherapy regimens.^404^ Overall, TTM is a low-toxicity, safe, and well-tolerated copper-chelating agent that has shown promising antitumor effects, particularly as an adjuvant treatment and in combination treatments.^155,166,280,396,397,404–407^ Additionally, serum ceruloplasmin levels serve as a biomarker to monitor and assess copper depletion, facilitating adjustments to drug dosages and treatment durations.^396,405^ However, in a study with 19 patients diagnosed with Hormone-Refractory Prostate Cancer, TTM monotherapy failed to delay disease progression.^408^ A possible reason for this could be that the anti-angiogenic effects of copper depletion by TTM may require a longer lead-in time to effectively inhibit tumor progression.^408^ Therefore, extending drug exposure time and designing studies to assess disease progression over time may contribute to optimizing treatment strategies.

ATN-224, a second-generation TTM analog, serves as an inhibitor of SOD1 in anticancer therapy.^409,410^ A phase I clinical trial involving 80 patients investigated the pharmacokinetics and pharmacodynamics of ATN-224.^411^ Results indicated that oral ATN-224 (330 mg/day) was well-tolerated in patients with advanced solid tumors. Compared to TTM, ATN-224 exhibited improved stability and a longer half-life, achieving a reduction in serum copper levels in 80% of patients within an average of 21 days (versus approximately 35 days for TTM). However, in addition to copper depletion-related anemia and neutropenia, ATN-224 also led to grade 3 fatigue after 14 days of treatment, which was dose-limiting and correlated with the dosage of ATN-224 and the rate of cp reduction.^411^ A phase II clinical trial on biochemically recurrent prostate cancer patients found that low-dose ATN-224 (30 mg/day) may exhibit biological activity.^412^ However, the clinical significance of prostate-specific antigen (PSA) dynamics in this population remains uncertain. Due to the lack of a clear dose-response effect, there are currently no plans for further development of ATN-224 for prostate cancer treatment.

D-pen is the first copper-chelating agent used for the treatment of WD and has been demonstrated to inhibit tumor proliferation and angiogenesis in preclinical models.^413^ For instance, D-pen inhibits LOX enzymatic activity by depleting copper levels, thereby suppressing angiogenesis and tumor progression in glioblastoma multiforme.^414^ Furthermore, D-pen enhances the cytotoxic effects of both radiotherapy and chemotherapeutic agents. Combining D-pen with oxaliplatin or cisplatin exhibits a synergistic cytotoxic effect on oxaliplatin-resistant cancer cells.^415^ The potential mechanism underlying this synergy is that D-pen upregulates the copper transport proteins hCtr1 and ATP7A, which share a transport system with platinum,^416^ thereby increasing intracellular platinum levels.^415^ Additionally, co-administration of D-pen with hydrogen peroxide metabolism inhibitors boosts the responsiveness of lung and breast cancer cells to radiation and carboplatin via H_2_O_2_-mediated oxidative stress.^417^ A Phase II clinical trial involving 40 patients with newly diagnosed glioblastoma treated with D-pen in conjunction with radiation therapy indicated that D-pen-induced copper deficiency was well tolerated. Drug-related myelosuppression, elevated liver function tests, and rashes were rapidly reversed with copper supplementation. Nevertheless, this anti-angiogenic strategy did not improve survival rates for glioblastoma patients compared to historical controls, with a median OS of 11.3 months and a median PFS of 7.1 months.^418^ Currently, a new clinical trial is underway to investigate the role of D-pen in enhancing radiosensitivity in recurrent head and neck cancer.

Trientine is an alternative copper-chelating agent clinically utilized for WD patients with D-pen intolerance.^419–421^ In preclinical tumor models, both Tientine and D-pen inhibited tumor progression; however, Tientine demonstrated a more significant inhibitory effect, primarily by suppressing angiogenesis and enhancing apoptosis within the tumors.^422,423^ Clinical trials of Trientine mainly focus on its application in platinum-resistant tumors,^424–426^ which re-sensitizes cancer cells to carboplatin by enhancing hCtr1-mediated platinum uptake.^427^ A preliminary clinical study involving five patients with platinum-resistant high-grade epithelial ovarian cancer receiving a combination of carboplatin and Trientine indicated that Trientine can partially reverse the resistance of cancer cells to platinum therapy, warranting further evaluation in larger studies.^424^ Another Phase I clinical trial that included 55 patients with advanced malignancies who had failed platinum treatment demonstrated that trientine, administered as a maximum daily divided dose of 3000 mg, can be safely combined with carboplatin FDA-approved doses (AUC 6), showing good tolerance.^426^ The longest duration of treatment recorded was 17 months, with no dose-limiting toxicities or treatment-related deaths observed. Compared to historical experiences with single-agent platinum therapy, no more severe adverse events were noted. Furthermore, patients with relatively low levels of hCtr1 expression may derive the greatest benefit from the combination treatment of carboplatin and trientine, suggesting a potential clinical prognostic implication.

TEPA has demonstrated tumor-suppressive effects in preclinical models of neuroblastoma.^20^ Specifically, TEPA inhibits the phosphorylation of STAT3 and EGFR, promotes the ubiquitin-mediated degradation of PD-L1, and significantly increases the infiltration of CD8 + T cells and natural killer cells, thereby enhancing antitumor immunity and slowing tumor growth.^20^ Additionally, TEPA has the potential to reduce radioresistance in HCC by chelating copper, functioning as a copper-dependent selective radiosensitizer.^236^ However, there are notable gaps in the clinical trial of TEPA for cancer therapy.

Nanomedicine delivery systems are designed to enhance cancer treatment by improving drug targeting and delivery efficiency while ensuring biocompatibility and biodegradability.^428^ These systems often utilize nanocarriers, such as liposomes, dendrimers, or polymeric nanoparticles, which can preferentially accumulate in tumor tissues via the enhanced permeability and retention (EPR) effect or by conjugating targeting ligands to specific tumor markers.^429,430^ Their biocompatibility minimizes toxicity to healthy tissues, while biodegradability allows for bodily clearance after therapeutic delivery. Advanced strategies, such as pH-responsive or enzyme-sensitive release mechanisms, optimize drug delivery by releasing therapeutic agents in response to the tumor’s unique microenvironment, thus maximizing efficacy and minimizing side effects.^431^ The clinical application of copper chelation in cancer treatment is limited by poor tumor-targeting capabilities, leading to associated toxicity or ineffectiveness. Therefore, nanoparticle-based delivery systems have been developed to optimize the therapeutic effects of small-molecule copper chelators. For instance, prostate-specific membrane antigen and glucose transporter GLUT1 targeting agents have been designed to deliver copper chelators specifically to prostate cancer and pancreatic cancer, respectively.^432^ Ismail M et al. successfully developed an actively targeted biomimetic nanoparticle (Ang-MNPs@(Dp44mT/Reg)) that can target and deliver the copper chelator Dp44mT to GBM lesions.^433^ These nanoparticles exhibit significantly enhanced active targeting capabilities and prolonged circulation time in the bloodstream, while markedly reducing drug-related side effects. This strategy not only effectively inhibits tumor growth and extends the survival of GBM model mice but also demonstrates negligible systemic toxicity, further validating its safety and potential for treating other central nervous system disorders.^433^ A mitochondria-targeted copper-depleting nanoparticle (CDN) demonstrates the ability to inhibit tumor growth and significantly extend survival in mouse models of TNBC.^434^ Compared to existing copper chelators, CDNs exhibit lower toxicity, as they preferentially deplete copper from the mitochondria of cancer cells rather than causing systemic copper depletion. Moreover, co-delivering copper chelators with other antitumor agents within the same nanoparticle carrier can harness the synergistic effects of different drugs to achieve multiple antitumor effects. For example, PTDH/R848 nanoparticles, which combine copper chelators with TLR7 and TLR8 agonists, can serve as a therapeutic agent for metastatic breast cancer by integrating anti-angiogenic and immune-activating mechanisms.^435^ Currently, various copper chelation-based nanomaterials have demonstrated antitumor effects and exhibit good biocompatibility. For instance, the novel adhesive injectable thermosensitive hydrogel loaded with small molecule copper chelator (SO-N)^436^ and the Imi-OSi nanocomplex with high selectivity and efficient copper-chelating capacity have shown promising results.^437^ However, no related drugs have yet entered clinical trials.

### Copper ionophores

Copper ionophores also referred to as cuproptosis-related drugs, typically form neutral, lipophilic complexes with copper, thereby increasing intracellular copper concentrations.^438^ A variety of copper ionophores have been developed as anticancer agents to promote cuproptosis, with ES and DSF being among the most extensively researched. In addition, several antimicrobial agents, such as clioquinol (CQ), 8-hydroxyquinoline (8-OHQ), NSC319726, and pyrithione, also function as copper ionophores.^439^ These compounds are instrumental in advancing the application of cuproptosis as a therapeutic strategy against cancer.

ES is a mitochondrion-targeting copper ionophore for cancer therapy.^283,440,441^ Previous studies have demonstrated that ES can induce copper-dependent apoptosis and ferroptosis by promoting the generation of ROS and facilitating the degradation of ATP7A, respectively.^22,282,442^ The discovery of cuproptosis has refined the specific cancer suppressor mechanism of ES, while enhanced mitochondrial metabolism increases the sensitivity of cancer cells to this agent.^26^ ES was initially developed as an adjunct chemotherapy agent for treating metastatic melanoma.^440^ Subsequently, sodium salt formulations of ES were developed for clinical trials, which could be used in combination with paclitaxel or as a monotherapy for various solid tumors and acute myeloid leukemia.^441,443–445^ Published data indicate that ES has a favorable safety profile but failed to show a favorable clinical response. A Phase I clinical trial involving patients with refractory solid tumors showed that the combination of ES and paclitaxel was well tolerated without increasing toxicity.^444^ In a Phase II trial conducted in patients with stage IV melanoma, the results indicated that the combination of paclitaxel and ES doubled the median PFS from 56 days to 112 days compared to paclitaxel alone, and reduced the risk of disease progression or death by 41.7%, extending the median OS from 7.8 months to 11.9 months.^441^ However, a large randomized double-blind Phase III trial involving 651 patients with advanced melanoma found that the combination of paclitaxel and ES did not achieve its primary endpoint of PFS.^446^ Interestingly, a post-hoc analysis indicated that baseline serum lactate dehydrogenase (LDH) levels could be a potential predictive factor for treatment response. In patients with low serum LDH levels, combination therapy improved median PFS by 1.6 months.^446^ Additionally, another Phase II clinical evaluation of the combination of ES and weekly paclitaxel for the treatment of recurrent or persistent platinum-resistant ovarian cancer, tubal cancer, or primary peritoneal cancer demonstrated good tolerability.^443^ However, the response rate was insufficient to support further research into this combination therapy.^443^

DSF, an FDA-approved aldehyde dehydrogenase (ALDH) inhibitor, exhibits multi-targeted anti-tumor activity across various cancer cell lines, making it a widely used anticancer agent.^447^ The toxicity of DSF is closely linked to its ability to promote intracellular accumulation of copper, significantly enhancing its anticancer efficacy when administered in conjunction with copper. The DSF/Cu complex targets multiple pathways, including inhibition of NF-κB, elevation of ROS levels, modulation of Npl4 aggregation, as well as induction of apoptosis, ferroptosis, and cuproptosis.^305,447–449^ Additionally, DSF possesses cytotoxic effects on ALDH-positive cancer stem cells which possess rapid self-renewal capabilities and strong tumorigenic potential and exhibit resistance to chemotherapy and radiotherapy.^450–452^ In preclinical models, the combination of DSF/Cu demonstrates potential synergistic effects when used in conjunction with established chemotherapy agents such as cisplatin, temozolomide (TMZ), gemcitabine, and doxorubicin.^453–455^ A Phase IIb multicenter, randomized, double-blind study involving 40 newly diagnosed NSCLC patients found that the addition of DSF to the combination therapy of cisplatin and vinorelbine was well-tolerated and prolonged median survival time from 7.1 to 10 months.^456^ Notably, two long-term survivors emerged in the DSF group.^456^ However, a Phase II clinical study involving 12 patients with recurrent and/or refractory germ cell tumors failed to achieve its primary endpoint.^457^ DSF showed limited activity in restoring cisplatin sensitivity, with only 2 patients achieving disease stabilization. The median PFS and OS were 1.4 months and 2.9 months, respectively. Current clinical trials suggest that DSF and DSF/Cu-based therapy have limited efficacy in cancer treatment. In a Phase I trial involving advanced solid tumor patients, participants received 250 mg of DSF with escalating doses of copper gluconate (2, 4, 6, or 8 mg of elemental copper), showing good overall tolerability.^458^ While five cases of grade 3 toxicity were observed (including anorexia, increased serum aspartate aminotransferase, increased serum alkaline phosphatase, fever, and fatigue), no dose-limiting toxicities were reported.^458^ However, a Phase II-III study with 88 patients suffering from recurrent glioblastoma, receiving 400 mg of DSF and 2.5 mg of copper daily in combination with alkylating chemotherapy, did not significantly improve the 6-month survival rate or median PFS.^459^ Additionally, the incidence of adverse events was notably higher in patients receiving the combination therapy compared to those on alkylating chemotherapy alone.^459^

Antimicrobial drugs inhibit microbial growth by increasing intracellular copper ion concentrations and can also function as copper ionophores to suppress cancer. CQ is an analog of 8-OHQ, both of which can induce apoptosis in tumor cells.^460,461^ Treatment with CQ in human tumor xenografts with high copper content also leads to cancer suppression, linked to proteasome inhibition in vivo. Further studies indicate that the proteasome inhibitory and growth suppressive effects of CQ and 8-OHQ on tumor cells require their ability to bind copper and facilitate its transport into cells.^462^ Additionally, CQ (at a concentration of 50 μM) induces the oxidation of the copper chaperone ATOX1, indicating its inactivation and subsequent impairment of copper transport. However, the inactivation of ATOX1, leading to disrupted cellular copper transport, is one of the mechanisms underlying the subacute bone marrow optic neuropathy neurotoxicity associated with CQ.^463^ Currently, the use of antimicrobial agents in the clinical treatment of cancer remains highly limited.

Cuproptosis, a novel copper-dependent mechanism of cell death, has garnered significant attention for its potent tumor-suppressive properties. However, its clinical application is limited by the low sensitivity of tumor cells, which can be attributed to several factors:^326,333^ insufficient copper ion concentration within mitochondria, high levels of GSH that chelate copper ions and inhibit copper-protein interactions, and the rapid clearance of copper ionophores like ES and DSF, which restrict copper uptake by tumor cells. Furthermore, relying solely on cuproptosis may not effectively control tumor growth. To overcome these challenges, nanomedicine delivery systems have emerged as a promising strategy to enhance copper accumulation and release at tumor sites, optimizing the therapeutic effects of cuproptosis.^464^ Research is focusing on synergistic cancer therapies that combine cuproptosis with various anti-tumor modalities, including chemotherapy,^465^ photodynamic therapy (PDT),^466^ photothermal therapy,^467^ immunotherapy,^326^ and gene therapy.^468^ For example, a study by Zhou et al. engineered a nanocarrier system (Au@MSN-Cu/PEG/DSF) for photothermally activated drug delivery, which utilizes the EPR effect to concentrate at tumor sites.^467^ Upon near-infrared laser exposure, localized heat triggers the release of copper (II), forming a cytotoxic complex that induces cuproptosis in tumors. Similarly, Xu et al. developed a nanoporous copper(I) 1,2,4-triazolate coordination polymer nanocarrier (GOx@[Cu(tz)]) that synchronizes therapies via cuproptosis, PDT, and starvation treatments by depleting glucose and GSH, thus enhancing tumor cell sensitivity.^466^ Additionally, Guo et al. created a ROS-sensitive polymer (PHPM) for co-encapsulation of ES and copper into nanoparticles (NP@ESCu), which, upon internalization, release these agents in response to elevated intracellular ROS. This approach not only induces cuproptosis but also increases PD-L1 expression, transforming “cold” tumors into “hot” tumors that respond to immunotherapy, significantly inhibiting tumor growth and activating a systemic anti-tumor immune response.^326^ Related therapeutic agents include T-HCN@CuMS,^469^ Cel-Cu NP,^379^ ES@CuO,^327^ Ce6@Cu NPs,^470^ ES-Cu-MOF,^471^ Cu@CDCN,^472^ mCGYL-LOx,^337^ AuTPyP,^333^ PDA-DTC/Cu,^473^ Cu-THBQ/AX,^383^ MACuS,^474^ CuO NPs,^475^ ZCA NSs,^476^ Cu2-xSe@cMOF,^477^ CSTD-Cu(II)@DSF,^465^and CuPEs@PApt,^478^ among others. Despite the lack of clinical trials, these findings highlight the promising clinical prospects of combining cuproptosis with various treatment modalities in cancer therapy.

### Detection of cuproptosis

Cuproptosis is a newly identified form of regulated cell death, yet biomarkers for its detection remain incompletely defined. In this context, we outline four key aspects for detecting cuproptosis based on existing literature and identify several biomarkers associated with this process. Currently, these biomarkers are primarily utilized to detect cuproptosis in cell lines or animal models, but they hold significant potential for advancing research in the field.

### Assessment of key cuproptosis-related genes or proteins

Cuproptosis is characterized by the oligomerization of DLAT, the decrease of Fe-S cluster proteins, and an increase in heat shock proteins, which can serve as indicators of cuproptosis. Two commonly employed methods for detecting DLAT oligomerization include non-reducing immunoblotting and confocal immunofluorescence imaging.^26,327,331,466,479–481^ Immunoblotting, immunofluorescence, and immunohistochemistry are typically used to analyze the expression of Fe-S cluster proteins (such as FDX1, LIAS, ACO2, ETFDH, NDUFV1, and NDUFS8) and heat shock proteins (HSP70).^326,482^ Notably, FDX1 is known to regulate the lipoylation of cellular proteins through direct interactions with LIAS.^298^ Consequently, the loss of FDX1 during cuproptosis is often accompanied by a reduction in the lipoylation of four mitochondrial enzymes: DBT, GCSH, DLST, and DLAT,^300,301^ among which lipoylated DLST and DLAT are also commonly detected via immunoblotting during cuproptosis. It is crucial to acknowledge that changes in the expression of these cuproptosis-related proteins are not specific to cuproptosis, as they are subject to complex transcriptional regulation and can be influenced by various factors, including cellular stress, signaling pathways, and genetic background. Therefore, while assessing these key proteins is essential, it is insufficient for researchers to definitively evaluate the occurrence of cuproptosis.

### Examination of the ultrastructure of subcellular organelles

Morphologically, cuproptotic cells exhibit mitochondrial shrinkage, cell membrane rupture, endoplasmic reticulum damage, and chromatin fragmentation.^483,484^ These alterations are critical for identifying cuproptosis within cellular contexts. Transmission electron microscopy (TEM) plays a vital role in elucidating changes in these subcellular organelles. For instance, Zhao et al. utilized TEM to demonstrate that the treatment of zebrafish embryos with copper nanoparticles and CuSO4 resulted in a reduction of mitochondrial inner membranes and a loosening of the endoplasmic reticulum structure.^200^ Similarly, Liao et al. observed that excess copper led to mitochondrial vacuolization, membrane destruction, and chromatin rupture in chicken liver cells.^213^ Further investigations using Bio-TEM revealed shrunken mitochondria with reduced or absent cristae and increased membrane density, confirming copper-induced mitochondrial toxicity.^338^ These findings suggest that cuproptotic cells are characterized by distinct ultrastructural changes in subcellular organelles, including mitochondria, endoplasmic reticulum, and nucleus. However, it is important to recognize that these morphological features may also overlap with other forms of cellular stress or death, particularly apoptosis.^200,213,215^ Therefore, while examining the ultrastructure of subcellular organelles can provide preliminary evidence for cuproptosis, additional confirmatory assays are necessary to specifically attribute these changes to cuproptosis.

### Copper levels assessment

Excess copper in the cytoplasm and subcellular organelles serves as a key inducer of cuproptosis. Thus, precise quantification and distribution mapping of copper in mitochondria, endoplasmic reticulum, nuclei, and cytoplasm are essential for researchers evaluating cuproptosis. Various techniques, including copper-specific fluorescent probes, colorimetric tests, and inductively coupled plasma mass spectrometry (ICP-MS), can be employed to measure copper levels.^485–488^ ICP-MS offers high sensitivity and accuracy in detecting trace amounts of copper ions, enabling researchers to identify subtle fluctuations in copper levels that may impact cellular metabolism and function.^489^ In colorimetric assays, copper ions in the sample react with a complexing agent to form a purple complex, allowing for indirect calculation of copper ion content.^490^ Given the well-established role of copper in mitochondria, copper-specific fluorescent probes and mito-tracker (a mitochondrial fluorescent probe) are commonly used to explore the relationship between copper and mitochondria.^338,385,491^ By employing mito-tracker alongside copper-specific fluorescent probes, researchers can effectively visualize the colocalization of copper ions within the mitochondrial matrix using fluorescence microscopy. In certain instances, researchers may also estimate copper levels indirectly by assessing the expression of copper importers (SLC31A1, also known as CTR1) and copper exporters (ATP7A and ATP7B),^114^ or by utilizing copper chelators such as tetrathiomolybdate or bathocuproine disulfonic acid to mitigate cuproptosis.

### Metabolic biomarker assessment

During cuproptosis, copper does not directly target the electron transport chain but rather affects components of the TCA cycle, making its metabolites critical indicators of this cell death process.^26^ Tsvetkov et al. demonstrated changes in various metabolites, including elevated levels of citrate, cis-aconitate, GDP, and sedoheptulose-7-P, alongside decreased levels of glutamate, α-ketoglutarate, succinate, fumarate, malate, and C5-Carnitine.^26^ The reduction in α-ketoglutarate and succinate partially contributes to the decreased expression of FDX1, whereas the implications of other metabolite changes and their predictive value for cuproptosis warrant further investigation. These metabolites can be quantitatively assessed using techniques such as liquid chromatography coupled with mass spectrometry or NMR spectroscopy for comprehensive metabolite profiling.

## Conclusion and perspectives

Copper functions as a double-edged sword within cells: it is an essential cofactor for numerous enzymes that facilitate tumor progression, yet its excess can lead to cuproptosis, resulting in cellular demise. In recent years, pharmacologically targeting copper and cuproptosis has emerged as a promising anticancer strategy. Therefore, exploring the foundational mechanistic aspects related to copper and cuproptosis could provide valuable treatment avenues for cancer. However, several challenges must be addressed in future research.

Firstly, monitoring copper levels within tumors and identifying biomarkers of cuproptosis in patients present significant challenges. Despite advancements in analytical techniques,^492^ including copper isotope measurement^493^ and chemical fluorescent probes,^494^ their application in cuproptosis-related research remains largely confined to cell lines or in vitro tissues. Progress in imaging technologies, such as copper-specific imaging agents,^495,496^ PET scans,^497–499^ and nanoprobes,^465^ could facilitate non-invasive monitoring of copper distribution in vivo. However, their sensitivity warrants improvement, and their safety in patients needs further clarification. Moreover, although cuproptotic cells are characterized by mitochondrial shrinkage, cell membrane rupture, endoplasmic reticulum damage, and chromatin fragmentation, these morphological changes overlap with those seen in apoptosis,^200,213,215^ complicating the issue. Thus, developing robust assays and biomarkers, along with standardized experimental protocols and methodologies, to accurately measure copper levels and assess cuproptosis in patients is imperative.

Secondly, the precise molecular mechanisms underlying cuproptosis remain poorly understood, despite its strong association with mitochondrial metabolic states. For instance, the mechanism by which mitochondrial respiration accelerates cuproptosis is unclear. One hypothesis posits that cells with active mitochondrial respiration may express higher levels of lipoylated enzymes, potentially leading to increased aggregation.^500^ However, there is currently no evidence indicating that tumor stem-like cells or certain drug-resistant cells with enhanced mitochondrial respiration exhibit elevated levels of lipoylated enzymes. Additionally, it remains uncertain why cell death caused by excess copper results in cuproptosis rather than established forms of cell death such as apoptosis or necroptosis. Further research is necessary to determine whether excess copper inhibits enzymes critical for these well-known cell death pathways. Furthermore, while mitochondria are known to play a crucial role in cuproptosis, it is uncertain if they are strictly required for the process or if cuproptosis can occur in mitochondria-depleted cells.

Thirdly, investigating the occurrence of cuproptosis under normal physiological conditions presents another challenge. Cuproptosis has been documented in several pathological conditions beyond Wilson’s disease, Menkes disease, and cancer. For example, Chen et al. demonstrated that cuproptosis occurs following myocardial ischemia-reperfusion injury, exacerbated by sleep fragmentation.^480^ Yang et al. reported that high glucose levels induce cuproptosis in human lens epithelial cells, which can be reversed by copper chelation.^501^ Additionally, SARS-CoV-2 infection has been found to reduce glutamine levels, leading to decreased glutathione levels, copper overload, and increased cuproptosis in immune cells, potentially exacerbating rapid tumor development.^502^ These findings underscore the prevalence of cuproptosis in various pathological conditions. Similarly, ferroptosis has been initially identified as contributing to the development of numerous diseases, including cancer, neurodegeneration, sepsis, ischemia-reperfusion injury, autoimmune disorders, and metabolic disorders.^503^ With the deepening of research, ferroptosis is identified to play a significant role under normal physiological conditions such as embryonic development and aging.^504^ Thus, it is speculated that cuproptosis must also occur under several normal physiological conditions, requiring further investigations.

Fourthly, despite numerous studies analyzing the connections between CRGs and various tumor characteristics, the lack of biological evidence and experimental validation presents an additional challenge. Many studies only indirectly demonstrate a link between cuproptosis and cancer,^363,447,505–507^ leaving unclear whether these genes play a direct role in this relationship or if they are influenced indirectly. Another unresolved question is whether other metabolic pathways, in addition to mitochondrial respiration, participate in cuproptosis. Additionally, while CTR1 and DMT1 are known receptors for copper uptake, recent work by Li et al. suggests that ZnT1, which exports zinc from cells, may also be involved in copper uptake and cuproptosis.^297^ This raises the possibility that other copper transporters mediating copper uptake or efflux warrant further investigation.

Lastly, it is crucial to identify patient groups likely to benefit from cuproptosis-based therapies, as certain cancers characterized by higher mitochondrial respiration, stem cell-like traits, or drug resistance have demonstrated better responsiveness to such treatments.^80^ A previous phase III clinical trial of ES in melanoma patients showed no clinical benefit;^446^ however, a post-hoc analysis indicated that patients with low plasma LDH levels may benefit from ES treatment, suggesting that LDH could serve as an important biomarker for screening patients who may respond favorably to copper-based therapy. A possible explanation for this is that lower LDH levels reflect a higher cellular dependency on mitochondrial respiration. Nevertheless, there is an urgent need to identify more reliable biomarkers to effectively screen potential beneficiaries. Furthermore, inhibitors of the proteasome or Wnt signaling pathways might synergize with copper ionophores in tumor therapy.^293,296^ Numerous studies suggest that cuproptosis-based therapies have the potential to enhance the efficacy of immunotherapies.^324,327,334,379–388^ Therefore, identifying effective combinational strategies for cuproptosis-based interventions is essential for future applications.

In summary, this review enhances our understanding of the molecular mechanisms and therapeutic landscape of copper and cuproptosis in cancer. Addressing the aforementioned challenges will not only deepen our comprehension of its role in cancer biology but also pave the way for the development of copper and cuproptosis-based therapies. We anticipate that intensive research efforts will facilitate the translation of the concept of cuproptosis from basic science into therapeutic reality.
